# Modality-general sensitivity of pupil responses to regularity violations

**DOI:** 10.3758/s13415-026-01423-3

**Published:** 2026-04-07

**Authors:** Hamit Basgol, Florian Raab, Peter Dayan, Volker H. Franz

**Affiliations:** 1 Department of Computer Science, University of Tübingen, Tübingen, Germany; 2 Experimental Cognitive Science, University of Tübingen, Sand 6, 72074 Tübingen, Germany; 3 The Graduate Training Centre of Neuroscience, University of Tübingen, Tübingen, Germany; 4 Max Planck Institute for Biological Cybernetics, Tübingen, Germany

**Keywords:** Pupil size, Arousal, Uncertainty, Visual regularities, Auditory regularities

## Abstract

Pupil dilation responses are reliable physiological markers of arousal in response to unexpected events. We investigated how these responses generalise across different sensory modalities by using transitions between regular and random sequences of visual dots and auditory tones. In Experiment 1, we investigated sequences of visual dots and found that (a) transitions from a regular sequence to a random sequence induced pupil dilations, (b) transitions from one regular sequence to another regular sequence also induced pupil dilations, and (c) transitions from a random sequence to a regular sequence did not reliably induce them. In Experiment 2, we replicated these findings, confirming their reliability and thereby generalizing the literature from the auditory to the visual modality. In Experiment 3, we directly compared pupil dilations in visual and auditory modalities. We observed strong cross-modal similarity in pupil sizes, particularly for transitions between regular and random sequences. We also decomposed the pupil size time series to approximate phasic pupil dilation events. While the patterns of dilation events were quite similar, differences between modalities in dilation size (but not in rates) occurred during transitions from one regular to another regular sequence. Overall, our findings suggest that pupil-linked arousal reflects inference of statistical structure and its violations, exhibiting substantial (albeit not perfect) similarity across modalities.

**Supplementary Information:**

The online version contains supplementary material available at 10.3758/s13415-026-01423-3.

Sensory input exhibits statistical regularities over multiple temporal and spatial scales. A wide range of studies has demonstrated that human observers track these statistical regularities (Barascud et al., 2016; Canale, 2022; Conway, 2020; Frost et al., 2015; Paavilainen, 2013; Sherman et al., 2020). Regularities exhibit a complex structure, leading observers to experience prediction errors of varying degrees across various time scales. In particular, low-probability, salient, and abrupt changes generate strong brain responses and highlight the requirement for learning and adaptation (Dayan & Yu, 2006; Jordan, 2024; Soltani & Izquierdo, 2019).

Several biomarkers have been associated with prediction errors, notably various electroencephalography (EEG) signatures. These include the Mismatch Negativity Response (MMN), an early EEG component (100–200 ms) elicited by a deviant stimulus within a regularly structured sequence. Although primarily studied in the auditory literature, MMNs are observed across all modalities, and concern the predictability of stimuli independent of modality of interest; and have been taken as reflecting hierarchical inferences across cortices (Duncan et al., 2009; Grundei et al., 2023; Lieder et al., 2013; Prete et al., 2022; Schröger et al., 2015; Sussman, 2005). MMNs are seen, for example, in audition (Grundei et al., 2023; Lecaignard et al., 2022), somatosensation (Grundei et al., 2023), and vision (Kremláček et al., 2016). Equally, the P3 component (300–500 ms) responds to prediction errors and has been observed across auditory, somatosensory and visual modalities (Lau et al., 2019; Duncan et al., 2009; Zhang et al., 2022), and multi-modal sequences (Grundei et al., 2023).

Critically, the brain’s response to violations exemplified by the MMN and P3 is also frequently accompanied by a physiological marker: pupil dilation, a type of pupil response characterised by an increase in pupil size (Alamia et al., 2019; Basgol et al., 2025; Bianco et al., 2020; Liao et al., 2016a, b; Planton & Dehaene, 2021; Zekveld et al., 2018; Zhao et al., 2019).

Pupil dilations are associated with activity of the locus coeruleus-norepinephrine system (the LC-NE system; de Gee et al., 2017; Glennon et al., 2019; Joshi and Gold, 2020; Joshi et al., 2016; Rylan et al., 2018; Peter et al., 2014; Reimer et al., 2016; Strauch et al., 2022) as well as of the basal forebrain-cholinergic system (the BF-ACh system; Lloyd et al., 2023). Such responses have long been investigated as reflecting computational processes in the brain (Basgol et al., 2025; Filipowicz et al., 2020; Rylan et al., 2018; Nassar et al., 2012; Pajkossy et al., 2023; Zhao et al., 2019), even when these responses are not directly relevant to task requirements or when the effect of task requirements on pupil size is statistically controlled (Alamia et al., 2019; Basgol et al., 2025; Liao et al., 2016b; Zhao et al., 2019).

The pupil dilates, for example, in response to the violation of simple, regularly structured visual or auditory stimuli by a deviant stimulus (Alamia et al., 2019; Liao et al., 2016b; Zekveld et al., 2018). Equally, the pupil also dilates in response to unexpected transitions from a regular sequence (structured by repeating a set of 50-ms-long tone pips) either to a random sequence (Zhao et al., 2019) or a different regular sequence (Basgol et al., 2025). The magnitude and the number of dilation events are modulated by the extent of violations introduced by these transitions (i.e., the change in surprise, Basgol et al., 2025). These results are consistent with the view that phasic pupil dilation responses reflect state transitions (Basgol et al., 2025; Bouret & Sara, 2005; Dayan & Yu, 2006; de Gee et al., 2017; Krishnamurthy et al., 2017; Nassar et al., 2012; Angela & Yu, 2012; Yu & Dayan, 2005). That is, a sufficiently strong environmental change, indicating a state transition, would elicit the LC-NE system activity associated with arousal, which in turn would reset cortical target circuits to enhance focus on novel information (Bouret & Sara, 2005; Dayan & Yu, 2006).

However, this previous research on pupillary responses to statistical changes in complex sequences has focused on audition. Growing evidence suggests a modality-specific advantage for audition over vision: the auditory system is more efficiently responsive to sequential patterns across time, whereas the visual system is better at patterns across space (Conway, 2020; Conway & Christiansen, 2005, 2009; Emberson et al., 2011; Ferguson et al., 2018; Frost et al., 2015; Hans et al., 1966; Gregg & Recanzone, 2009; Robinson & Sloutsky, 2007). These differences, among others (Conway, 2020; Frost et al., 2015), have raised questions about the domain-generality of sequential processing. Conversely, emerging evidence, including EEG biomarkers (MMN and P3; Grundei et al., 2023), and the engagement of overlapping brain networks across sensory modalities (Planton & Dehaene, 2021), suggests that domain-general mechanisms may underlie responses to prediction errors, potentially operating in concert with modality-specific processes (Conway, 2020; Frost et al., 2015). Since vision is also capable of processing and learning statistical relationships within sequences (Conway, 2020), we investigated generalization of pupillary responses to statistical changes in complex visual sequences, and compared the responses across visual and auditory modalities.

## Purpose of the study

Bringing together these threads, we sought to investigate pupil dynamics, as a proxy of computational processes in the brain. We created a visual version of a rapid tone presentation paradigm (Barascud et al., 2016; Southwell & Chait, 2018; Southwell et al., 2017; Zhao et al., 2019) that we have also previously employed (Basgol et al., 2025). In this new visual version, a short-lived white dot appeared on a two-dimensional (2D) screen, moving across predetermined positions in either a regular (repeating trajectory) or random pattern. We employed three types of transitions between these patterns: random to regular, regular to random, or regular to a novel regular pattern. While transitions from random to regular patterns lead to the emergence of regularities (increasing predictability and reduction of surprise), those from regular to random or to novel regular patterns lead to their violations (reducing predictability and increasing surprise either continuously or momentarily, Barascud et al., 2016; Basgol et al., 2025; Zhao et al., 2019).

As for the case of audition, we isolated spontaneous processing of visual regularities and minimised the influence of decision-making and motor responses on pupil size (Privitera et al., 2010; Simpson, 1969), by instructing participants to perform the incidental task of detecting a target (e.g., a gap or a shape). This was to ensure that they kept their attention on the sequences.

First, we conducted two experiments, Experiments 1 and 2, to examine whether observations of pupil size from the auditory modality can be extended to the visual modality, with violations, but not the emergence, of visual regularities eliciting pupil dilation responses (Basgol et al., 2025; Zhao et al., 2019). The experiments differed in their incidental tasks: detecting a gap (Experiment 1, mirroring previous auditory studies; Basgol et al., 2025; Milne et al., 2021; Zhao et al., 2019) or detecting a brief shape change (Experiment 2).

Then, in Experiment 3, we compared pupil responses to visual and auditory regularity violations. Here, we built on previous studies that compared auditory and visual modalities (Stefania et al., 2018; Klingner et al., 2011; Liao et al., 2016b) by presenting transitions with varying degrees of statistical change.

## Experiment 1

In this experiment, we investigated whether we could extend previous auditory results, which demonstrate that regularity violations lead to pupil dilation responses (Basgol et al., 2025; Zhao et al., 2019), to the case of vision.

### Method

The raw data, processed data, and analysis scripts associated with studies in this manuscript are available on Zenodo (10.5281/zenodo.18613650). Unlike other experiments in this paper, this experiment and associated analyses were not preregistered. However, the analysis plan closely followed our previous study with auditory stimuli (Basgol et al., 2025).

#### Participants

Fifteen participants contributed to the study. We dropped the data of one participant due to a high number of blinks and excessive data loss, resulting in 14 participants (11 female, one diverse, M$_\text {age}$: 23.78, SD$_\text {age}$: 3.25). The number of participants was comparable to experiments investigating the effect of auditory regularity violations (Zhao et al., 2019). Participants were mostly university students and were compensated with either course credit or 10 EUR per hour. The experiment took approximately 1.5 hours.

**Fig. 1:**
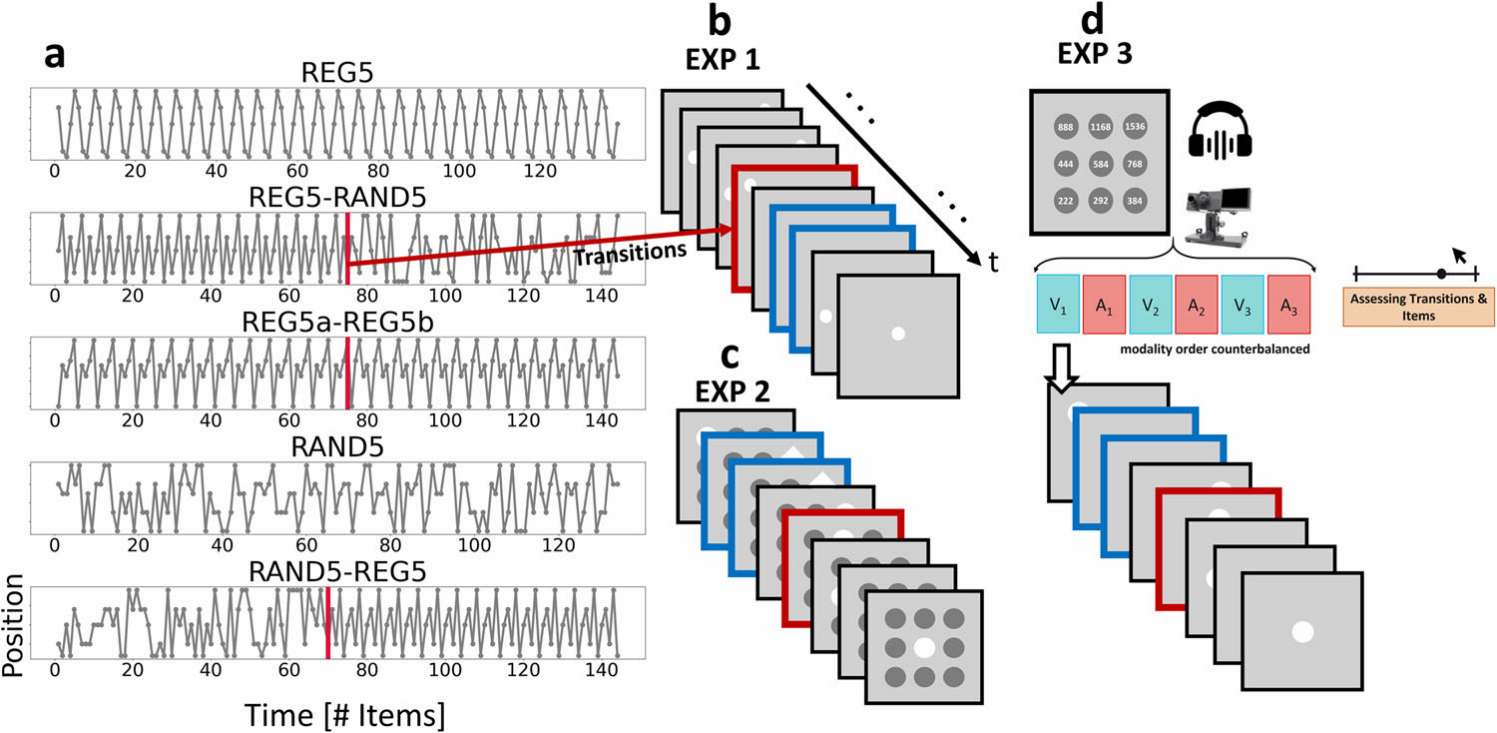
Conditions and experiments. **a** An example set of visual stimuli. The x-axis represents time, and the y-axis represents the possible positions on a visual display of a white dot, as shown in (b, c, d). Note that 2D positions were flattened for a one-dimensional representation on the y-axis. Red vertical lines in (a) and red borders in (b, c, and d) indicate transitions between visual patterns (although the timing of transitions was jittered in experiments). RAND5: Five positions were randomly chosen from a set of 20 possible positions and shown in random order. REG5: Five positions were also randomly selected from the same position pool (without replacement), but these were presented repeatedly in a fixed, consistent order. Both RAND5 and REG5 served as control conditions, as they did not involve any statistical transition. RAND5-REG5: A sequence began with a RAND5 pattern and then transitioned into a REG5 pattern. REG5-RAND5: A transition occurred from a REG5 pattern to a RAND5 pattern. REG5a-REG5b: The sequence transitioned from one REG5 pattern to a different REG5 pattern. Therefore, REG5-RAND5 and REG5a-REG5b lead to violations of the previous regularity, whereas RAND5-REG5 lead to the emergence of new regularities. Note that we controlled for the introduction of new positions after transitions. Experiment 1 included all conditions; Experiments 2 and 3 included REG5, REG5-RAND5, and REG5a-REG5b. Pattern and trial durations differed across experiments. **b** Experiment 1 used a 2D display (4 rows x 5 columns). In this task, participants were required to detect gaps in the sequences (marked by blue borders). **c** Experiment 2 used a 3 x 3 display, where possible positions of the moving white dot were marked with a grey patch. Instead of a gap, participants were instructed to detect a white diagonal shape (marked by blue borders). **d** In Experiment 3, we compared pupil responses to visual and auditory regularity violations, and therefore included the presentation of visual and auditory sequences. Visual sequences were presented as the movement of a dot (without reference locations), similar to Experiments 1 and 2, whereas auditory sequences consisted of tones. Sequences from each modality were presented in separate blocks. Participants first detected gaps, and then they evaluated the items, which consisted of sequences, in terms of similarity and the saliency of transitions between patterns. Coloured borders (red, blue, and black) are drawn only for visualisation purposes

#### Materials

Previous studies generated random (RAND) and regular (REG) patterns by sampling a number (*n*) of items from a pool of 20-items (Barascud et al., 2016; Basgol et al., 2025; Southwell & Chait, 2018; Southwell et al., 2017; Zhao et al., 2019; Milne et al., 2021). For a RAND*n* pattern, *n* items were randomly chosen from the pool (with replacement), and were then randomly resampled (with replacement) until the desired total sequence length was reached. In contrast, for a REG*n* pattern, *n* items were again randomly chosen from the pool (with replacement), but were then presented repeatedly in the same order.

RAND and REG patterns shared the same low-level features but differed in their statistical properties. When trials involved a transition from one pattern to another (e.g., RAND*n*-REG*n* or REG*n*-RAND*n*), the same positions were used for both sequence types to avoid a confound with the novelty of items (Zhao et al., 2019; Basgol et al., 2025).

We sampled REG5 and RAND5 patterns to generate baseline trials. However, in our work, we used sampling without replacement to preserve complexity in REG*n* patterns. Trials involving RAND5-REG5 were used to investigate the effect of the emergence of a regularity on pupil size. Trials involving the transition REG5-RAND5 were used to investigate the effect of regularity violation. We also included a transition between different regularities, REG5a-REG5b (which were different but used the same 5 items; see Fig. 1a). Comparing REG5-RAND5 with REG5a-REG5b allowed us to compare the effects of violations that later evolved into regularities.

In earlier versions of this paradigm, the items were short, 50 ms tones (Barascud et al., 2016; Basgol et al., 2025; Southwell & Chait, 2018; Southwell et al., 2017; Zhao et al., 2019; Milne et al., 2021). In our current visual analogue of this paradigm, the items were short-lived white dot presented at positions in a 2D display (a grid with 4 rows x 5 columns; see Fig. 1b). Thus, regularities were structured by the spatio-temporal trajectory of the dot’s position, rather than by its identity (such as its colour or shape, see similar paradigms; Conway & Christiansen, 2005; 2009; Simon et al., 2016). This type of display allowed us to control for two potential sources of noise. That is (a) we minimised luminance-driven effects on pupil, and (b) kept the working memory load low for tracking the statistics across items, helping the regularities pop-out (Conway & Christiansen, 2009; demonstrating superior statistical learning for spatial position over identity).

Each participant observed 210 trials of randomly generated visual sequences (30 REG5-RAND5, 30 REG5a-REG5b, 60 REG5, 60 RAND5-REG5, and 30 RAND5). The length of trials varied from 6 to 8 s, and transitions occurred between 3.25 and 4 s after the sequence onset with a granularity of 0.25 s. REG5 presentations consisted of 24-32 repetitions of the same visual sequence (120-160 items in total), and the REG5 sequences in REG5-RAND5 and REG5a-REG5b were violated after 13-16 repetitions (65-80 items in total). Since visual regularities might have required more time to be recognised than auditory regularities, we increased the trial durations over those used in earlier auditory studies (Zhao et al., 2019; Basgol et al., 2025). Inter-trial intervals were 4 s with a 1-s feedback.

A gap detection task was used to maintain participants’ attention to visual sequences without requiring them to search for the transitions between patterns. This required participants to detect possible 0.4 s gaps, which occurred on 20% of trials and at any time between 0.5 s post-onset and pre-offset. Note that 0.4 s is approximately three times longer than the gap duration in earlier auditory research (0.15 s for RAND and 0.1 s for REG patterns; (Zhao et al., 2019; Basgol et al., 2025). We chose this duration based on pilot experiments, in which participants had difficulty detecting visual gaps less than 0.4 s. Each condition involved the same proportion of gaps.

The main experiment consisted of six blocks, each containing trials from different conditions. Participants then completed a short control block. Trials in this control block were REG5 or RAND5, and in 50% of the trials, a single tone pip was also presented (0.5 s, 1000 Hz). This control block was designed to assess whether the visual properties of the experiment alone induced a ceiling effect in pupil size. If such a ceiling effect were present, we would expect no pupil dilation response to the tone pip, and the absence of responses would have been due to the inability of the pupil to dilate further.

The visual stimuli were presented using a ViewPixx/3D System (screen diagonal: 24 inches, 60.96 cm). Participants were provided a RESPONSEPixx connected to the monitor and could respond by choosing one of the five buttons. Their right eyes (unless specified) were tracked using an EyeLink 1000 system (SR Research) at a sampling rate of 1000 Hz.

#### Procedure

Participants sat in a luminance-controlled, dimly lit room (5 cd/m^2^). They were instructed to place their chin on a chin-rest to maintain a fixed distance between the eye and the monitor (50 cm), and the eye tracker was positioned below the monitor.

Participants were shown an example of a trial that included a gap and then attended a brief practice session. They were instructed to monitor the sequences for gaps and respond with a button press. To minimise the influence of gaze position and retinal luminance changes (due to shifts in the white dot’s position) on pupil, participants were instructed to fixate on the cross at the centre of the screen (or the centre of the screen if the cross was absent).

The six blocks of the main experiment were separated by an optional 3-minute break. Before each block, a calibration phase was conducted to align gaze location data recorded by the eye tracker. Following the main experiment, participants completed the control experiment.

### Analysis

#### Analysis of behavioural performance

We transformed participants’ hit and false alarm rates using an arcsine function for subsequent parametric and Bayesian statistical analyses (Zhao et al., 2019; Basgol et al., 2025).

#### Analysis of pupil responses

Pupil area in the right eye was recorded at a sampling rate of 1000 Hz. Trials with a gap or a button press were excluded from analysis to eliminate potential effects of the gap or motor responses on pupil (Privitera et al., 2010; Simpson, 1969). For RAND5-REG5, REG5-RAND5, and REG5a-REG5b conditions, pupil data were segmented from 1 second before to 3 seconds after transitions. For REG5 and RAND5 conditions, dummy transitions corresponding to the times of real transitions on other trials were randomly picked.

Pupil size was reconstructed by piecewise cubic interpolation when there were complete or partial blinks. Trials with 20% missing data due to blinking, and those involving missing data after interpolation, were excluded from the analyses. Due to excessive trial loss (70%), one participant was excluded.

We controlled the effect of gaze on pupil size by analysing participants’ gaze direction: none of the participants’ mean gaze locations exceeded the group mean by more than three standard deviations. As a control analysis, we also quantified gaze stability for visual trials. For each participant, we computed their mean gaze position and determined trials in which the mean gaze deviated by more than three standard deviations from this participant-specific mean. No trial met this criterion; therefore, no data were excluded.

After smoothing the data with a 150 ms Hanning window, pupil size was z-scored by computing the mean and standard deviation separately for each participant and block. Baseline correction was performed by subtracting the average pupil size from the 1 s interval before each real or dummy transition, allowing us to assess the impact of transitions on pupil size. A similar analysis pipeline was used to evaluate the effect of the tone pip on pupil size, with data segmented from 1 s before to 3 s after its onset. Pupil size in the no-transition control conditions (REG5 and RAND5) was also analysed over the whole trial (see Supplementary Figure S1).

Upon aggregating the data, we compared conditions using a non-parametric permutation procedure that controls the family-wise error rate. This procedure employed 5,000 permutations, with the null distribution generated by randomly sign-flipping participants’ average pupil size differences between the transition conditions with their corresponding no-transition control (implemented in MNE-Python, using an initial cluster-forming threshold of $p < .05$; Gramfort et al., 2013; Larson et al., 2022; Maris and Oostenveld, 2007). We transformed *t*-values to Bayes factors (BF$_{10}$ or BF$_{01}$ for estimating the evidence of the null hypothesis) with Jeffreys, Zellner, and Siow (JZS) prior using a Cauchy scale factor of 0.707 (Pingouin package in Python, Vallat, 2018). *p* values associated with time-independent comparisons (i.e., mean-based tests) were corrected using the Holm-Bonferroni method, and corrections were noted where applicable.

#### Analysis of pupil events and their magnitudes

The standard analysis in pupillometry involves averaging event-related pupil responses across trials. However, this appro-ach may distort peak amplitudes and response latencies (Fink et al., 2024), similar to challenges observed in other biological signals such as unctional magnetic resonance imaging (fMRI) and EEG (Guy et al., 2021; Wang et al., 2021). In our experience, two key issues can contribute to this distortion: (a) variability in pupil response times across trials and (b) reductions in pupil size as an orientation response to an event.

One solution to these issues is to extract small pupil events, such as micro-dilations and constrictions, from continuous pupil size measurements. These events are relevant because, as a proxy of phasic pupil response, they are associated with the activity of the LC-NE system (Joshi et al., 2016), especially when their magnitudes are high (Megemont et al., 2022). We identified these events by detecting the moments when the pupil size increased or decreased, analysing the slopes of pupil size (computed using the np.gradient function from numpy in Python, Basgol et al., 2025; Joshi et al., 2016; Megemont et al., 2022; Milne et al., 2021; Zhao et al., 2019).

Transitions can elicit saccadic eye movements, which may, in turn, influence pupil size (Winn et al., 2018) and lead to an overestimation of event rates. We addressed this potential confound in several ways: Participants were instructed not to follow the dots with their gaze, and gaze-related control analyses described in the previous section were applied. Furthermore, to minimise overestimation, we restricted our analysis to pupil events lasting longer than 300 ms, a threshold that was also adopted in previous studies (see Basgol et al., 2025; Zhao et al., 2019).

**Fig. 2:**
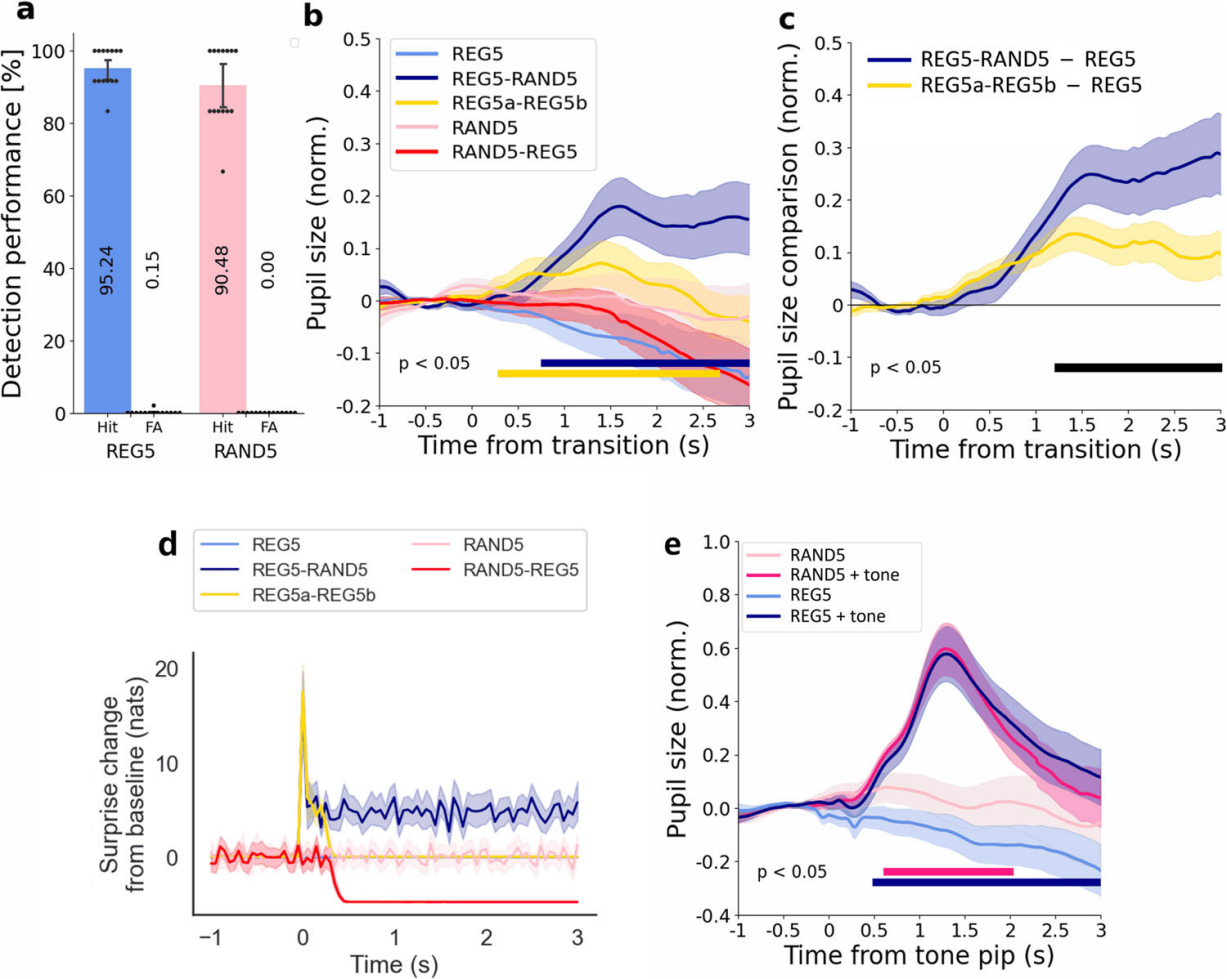
Gap detection performance and pupil responses in Experiment 1. Results of Experiment 1. **a** Hit and false alarm rates, Hit and FA, respectively, for the gap detection performance. Single points correspond to the performance of individual participants. Error bars indicate bootstrapped 95% confidence intervals. **b** Baseline corrected pupil size. Time 0 s marks real or dummy transitions to the next pattern. Regularity violations (REG5-RAND5, REG5a-REG5b) evoked pupil dilation responses, but the emergence of regular patterns (RAND5-REG5) did not. **c** Difference between regularity violation conditions and corresponding no-transition conditions. Even though both types of violations led to a pupil size increase, the REG5-RAND5 condition was associated with greater pupil dilation than REG5a-REG5b, which is indicated by the black horizontal line. **d** Results of surprise analyses. Surprise values were estimated using a hierarchical Chinese restaurant process (HCRP) sequence model (Éltetö et al., 2022), fitted to the sequence of trials experienced by each participant during the experimental session. The average surprise from the 1-second interval preceding the transition (from -1 to 0 s) was calculated and subtracted from the values for each trial to correct for baseline. **e** Pupil dilation responses to tone pips. In (b, c, and e), coloured horizontal lines mark time clusters with cluster-level $p < .05$ relative to no-transition control conditions. Shaded areas indicate the between-participants’ SEMs

We applied a 500-ms sliding window (based on each participant’s mean across trials) to the event data, separately for each participant and condition. Events were encoded as one at the time point at which they occurred, and their magnitude was defined as the magnitude difference from the preceding zero crossing, which served as the baseline. This analysis produced two distinct time series: one reflecting the count of events, and the other also reflecting their size. Baseline correction followed the same procedure as the pupil size data. We discuss this analysis in Experiment 3 (see Supplementary Figure S6).

#### Analysis of the statistics of the sequences

We analysed the surprise afforded by transitions in the sequences using a hierarchical Chinese restaurant process (HCRP) sequence model (Teh, 2006; Éltetö et al., 2022). We adopted this model to ensure a domain-general account of sequence learning that does not rely on modality-specific assumptions. Furthermore, this model is agnostic to the unexpected and expected certainty that underpins the design of the experiment (Yu & Dayan, 2005), which makes its predictions unbiased relative to our hypotheses. We calculated the surprise for all experiments. For the sake of clarity, we present only the results of Experiment 1. The other experiments showed a similar pattern of results (see Supplementary Section S3).

### Results

#### Behavioural performance

Participants performed similarly across conditions (see Fig. 2a). Paired two-sided *t*-tests revealed no significant difference in hit rates between REG5 and RAND5 (REG5 = 95.2, RAND5 = 90.5, $BF_{10}$ = 0.374, *p* = .399, 95% CI = [-0.140, 0.320], $d_z$ = 0.327) nor false alarm rates (REG5 = 0.146, RAND5 = 0, $BF_{10}$ = 0.413, *p* = .336, 95% CI = [-0.0017, 0.0046], $d_z$ = 0.277), indicating that the task was similarly challenging across conditions.

#### Pupil responses

The emergence of visual regularities (RAND5-REG5) did not elicit significant pupil dilation (compared to RAND5, see Fig. 2b). By contrast, violations of visual regularities (REG5-RAND5 and REG5a-REG5b) did lead to pupil dilation (compared to REG5, Fig. 2b). These results are consistent with previous findings in the auditory domain (Basgol et al., 2025; Zhao et al., 2019). To isolate the effect of transitions, we subtracted pupil size in the REG5 condition from pupil size in REG5-RAND5 and REG5a-REG5b conditions (see Fig. 2c). The increase in pupil size in REG5a-REG5b (approximately 0.15) was smaller than in REG5-RAND5 (approximately 0.25).

Pupil size has been shown to scale with the amount of surprise (Basgol et al., 2025). We estimated surprise profiles of visual transitions using an hierarchical Chinese restaurant process model (Teh, 2006; Éltetö et al., 2022) that was agnostic to the difference between unexpected and expected uncertainty (see Figure 2d). The model suggests that the surprise for the REG5-RAND5 condition upon transition was transient and sustained (see dark blue line in Fig. 2d); in contrast, the surprise for REG5a-REG5b was transient (see orange line in Fig. 2d). The surprise for RAND5a-REG5b reduced after the transition (see red line in Fig. 2d).

In the control block, participants were presented with a short tone pip, which also elicited pupil dilations (see Fig. 2e), indicating that merely performing a visual task does not prevent the pupil from responding to an arousing stimulus, consistent with previous findings (Marois & Vachon, 2018).

### Discussion

Visual regularity violations in the REG5-RAND5 and REG5a-REG5b conditions resulted in pupil dilations; in contrast, the emergence of visual regularities (RAND5-REG5) did not lead to a considerable increase in pupil size. These results generalised previous findings in the auditory domain (Basgol et al., 2025; Zhao et al., 2019). The REG5-RAND5 condition elicited a greater increase in pupil size compared to the REG5a-REG5b condition, reflecting the sustained surprise response (see Fig. 2d).

The lack of a pupil dilation response to the emergence of visual regularities (see Fig. 2b) is notable, specifically for vision, given that temporal regularities at a spatial location have been shown to guide attention of participants, even when such regularities do not provide any information about the task (Zhao et al., 2013; Zhao and Luo, 2017, but see Alamia and Zénon, 2016). This has been interpreted as a task-irrelevant attentional bias towards visual regularities (not observed for auditory regularities, Southwell et al., 2017). Pupil responses reflect the activity of attentional networks (Strauch et al., 2022); therefore, it needs to be examined whether and how regularities influence attention, and whether pupil responses (including constrictions) are sensitive to such regularities (Binda et al., 2025).

## Experiment 2

We next administered a pre-registered version of Experiment 1 to ensure that the difference between the two conditions (REG5-RAND5 vs. REG5a-REG5b) arises from transitions between patterns rather than the experimental structure. We therefore adjusted both the task and the stimulus display: (a) we used a 3 x 3 grid, limiting the display to nine locations. We expected that this would enable participants to monitor dot positions more effectively. Following this change, the dot size was set to 1° of the visual angle, (b) we marked the locations on the reference grid where the white dot could appear, and (c) participants were instructed to detect brief changes to the shape of the white dot.

### Method

We preregistered this study using AsPredicted (https://aspredicted.org/db33-5vg8.pdf). We highlight deviations from the preregistration and indicate exploratory analyses where applicable.

**Fig. 3:**
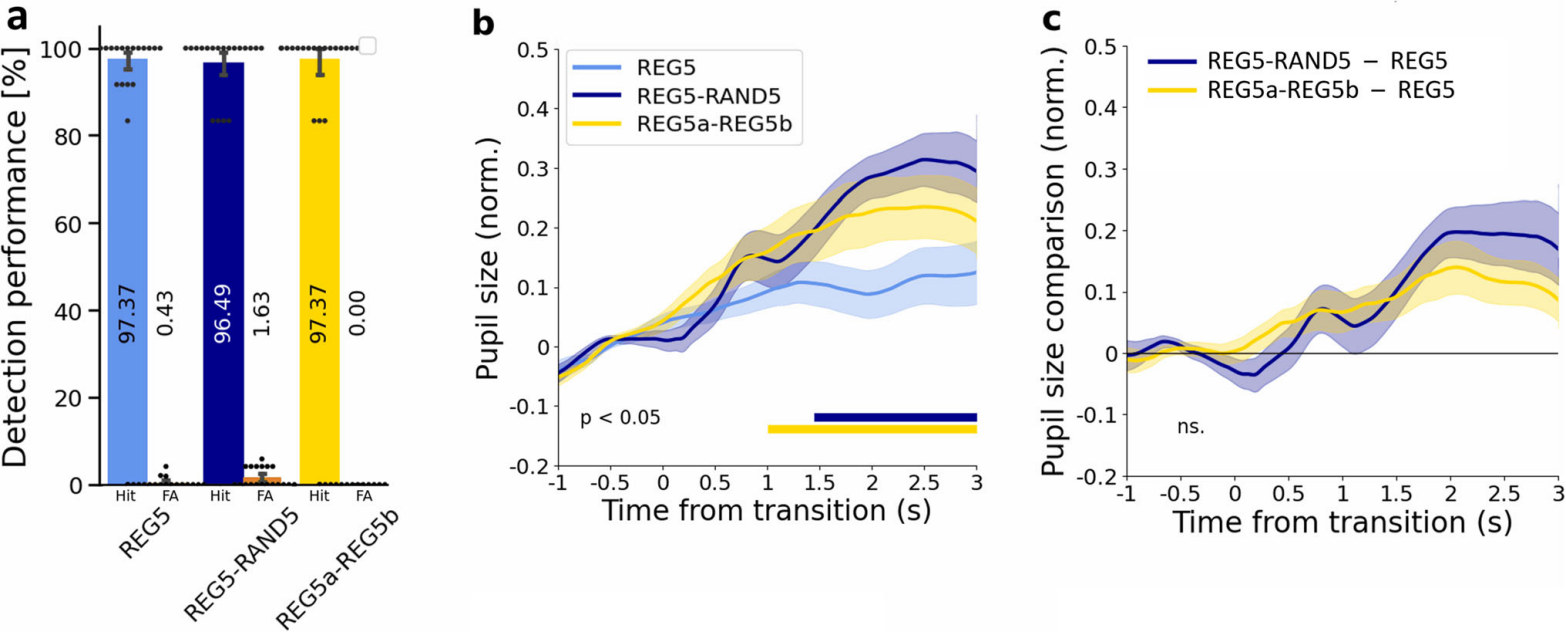
Shape detection performance and pupil dilation responses in Experiment 2. Results of Experiment 2. **a** Hit and false alarm rates, Hit and FA respectively, for the shape detection performance. Single points correspond to the performance of individual participants. Error bars indicate bootstrapped 95% confidence intervals. **b** Baseline corrected pupil size. In line with Experiment 1, pupil dilation responses were induced by regularity violations (REG5-RAND5, REG5a-REG5b). **c** Difference between regularity violation conditions and corresponding no-transition conditions. Both types of violations led to an increase in pupil size. Error bars in (a) and shaded areas in (b and c) indicate the between-participant SEMs. Coloured horizontal lines indicate regions where cluster-level statistics $p < .05$. The abbreviation ns. indicates non-significance

#### Participants

We calculated the number of participants based on the effect size we found in Experiment 1. To estimate the effect size, we first identified peak pupil dilation responses in the grand average for REG5-RAND5 and REG5a-REG5b. Then, using this information, we calculated the difference in amplitude between the REG5-RAND5 and REG5a-REG5b conditions and the corresponding control condition, REG5, for each participant. Note that calculating the effect size this way is more conservative than calculating the effect size based on time-series of pupil sizes, as the grand average does not consider pupil response differences across individuals.

The effect size of Experiment 1 was ${d}_\text {z} = 0.75$ and ${d}_\text {z} = 1$ for REG5a-REG5b and REG5-RAND5, respectively (based on a one-tailed student *t*-test). Using G*Power (Faul et al., 2007), with ${d}_\text {z} = 0.75$, we found that N = 18 is enough for an adequately powered study with 1 - $\beta$ = 0.92 and $\alpha$ = 0.05.

To improve upon Experiment 1, we measured participants’ visual acuity using the Freiburg Vision Test (the FrACT) and included only those with a visual acuity better than or equal to 0.3 logMAR (i.e., within the normal vision range; Bach, 2006). We excluded two participants due to eye tracker calibration problems, as inaccurate gaze measurements could affect pupil size estimation. Including their data did not alter the overall pupil response pattern. We included the data of 19 participants in our analyses (14 female, M$_\text {age} = 22.7$, SD$_\text {age} = 4.21$, mean M$_\text {logMAR} = -0.14$, SD$_\text {logMAR} = 0.13$). Note that this number is one more than specified in the preregistration; however, we retained this participant, as its inclusion did not change the overall pattern of statistical results. The experiment lasted 1.5 hours.

#### Materials

Each participant observed 120 trials of randomly generated visual sequences (30 REG5-RAND5, 30 REG5a-REG5b, and 60 REG5 as a baseline). The length of trials was 8 s, and transitions occurred between 3.5 s and 4 s after the sequence onset with a granularity of 0.25 s. REG5s consisted of 32 repetitions (160 tones), and REG5s in REG5-RAND5 and REG5a-REG5b were violated after 14-16 repetitions (70-80 tones). Participants were instructed to detect a brief change of the white dot into a diamond lasting 0.1 s.

#### Procedure

The procedure for Experiment 2 was similar to that of Experiment 1, with a few key differences: we assessed participants’ visual acuity using the FrACT (Bach, 2006), participants required to achieve an 85% hit rate during the practice session, and they filled out a questionnaire after the experiment.

### Analysis

#### Analysis of behavioural performance

We preprocessed the data as specified in the preregistration; as a result of the exclusion criteria, 36 trials (approximately $2\%$) were excluded from the analysis.

### Results

#### Behavioural performance

Participants performed similarly across conditions, with hit rates around 96% and false alarm rates between 0-2% (see Fig. 3a). Repeated measures ANOVA did not reveal a significant effect for hit rates, *F*(2, 36) = 0.13, *p* = .870, $\eta _{p}^{2}$ = 0.007. In contrast, the numerically small difference, $\approx 1.5\%$, in the REG5-RAND5 condition led to a statistical difference across conditions, *F*(2, 36) = 6.84, *p* = .003, $\eta _{p}^{2}$ = 0.28. The REG5-RAND5 condition received more false alarm responses than the REG5a-REG5b condition (*p* = .015, BF_10_ = 9.26, *d*_z_ = 1.04), but not more than the REG5 condition (*p* = .126, BF_10_ = 1.61, *d*_z_ = 0.68).

#### Pupil responses

The REG5-RAND5 and REG5a-REG5b conditions evoked pupil dilation responses (see Fig. 3b). Although average pupil size in REG5a-REG5b was smaller than in REG5-RAND5, the test did not provide sufficient evidence for a difference (pupil size in the REG5 condition was subtracted; Figure 3c).

### Discussion

Pupil responses show a similar pattern across visual and auditory modalities (Zhao et al., 2019; Basgol et al., 2025), providing evidence for domain-general responses to prediction errors (see Supplementary Figure S5 for comparisons of all experiments; Conway, 2020; Grundei et al., 2023; Sabio-Albert et al., 2025; Planton and Dehaene, 2021). However, we have so far only compared pupil responses to violations of visual regularities with existing results in the literature (Basgol et al., 2025; Zhao et al., 2019). These comparisons were, therefore, qualitative.

Previous studies have quantitatively compared the effects of presentation modality on pupil size by manipulating cognitive effort (Klingner et al., 2011; Lisi et al., 2015; Stefania et al., 2018); however, for unexpected events, modalities have mainly been studied using a limited, simple set of stimuli that lack variance (Zekveld et al., 2018; Liao et al., 2016b). We therefore conducted Experiment 3 to get a more comprehensive comparison between modalities.

## Experiment 3

In Experiment 3, we presented participants with sequences from visual and auditory modalities. These sequences shared the same conditional relationships across items in time, but they differed in presentation modality (i.e., tones for auditory stimuli and dot locations for visual stimuli, a standard method used in the literature; see Conway & Christiansen, 2005); they were, therefore, isomorphic.

Differences in pupil response dynamics may arise from modality-specific characteristics. Prior work has shown a linear relationship between subjective judgements of saliency and pupil dilation (Liao et al., 2016a). Therefore, to further investigate cross-modal effects, we compared pupil responses based on participants’ ratings of saliency (see Supplementary Figure S10).

We hypothesised that violations of regularities would increase pupil size. Additionally, we expected isomorphic transitions to evoke similar pupil responses, as reflected in both time-series and scalar pupil measures. We also hypothesised that pupil responses across modalities would present similar relationships with saliency ratings.

### Method

This experiment was preregistered on AsPredicted (https://aspredicted.org/v3wq-bszn.pdf). We highlight any deviations or exploratory analyses, when applicable.

#### Participants

We used the effect size from Experiment 1 (*d*_z_ = 0.75) to determine the required number of participants (N in short) for the effect of transitions, as the current experiment resembles Experiment 1 (in terms of visual display and task). Using G*Power (Faul et al., 2007), we found that a sample size of N = 20 would result in a power of 1-$\beta$ = 0.94 with $\alpha$ = 0.05.

We sought to test correlations of pupil responses across modalities. For this test, we planned to compute a Pearson correlation coefficient for each participant, and compare coefficients to zero using a one-tailed *t*-test. We therefore conducted another power analysis, using an effect size of $r = 0.2$ ($SD = 0.2$). This effect size aligns well with the average effect size reported in psychology (David et al., 2019) and is between a small ($r = 0.1$) and medium ($r = 0.3$) effect size (Cohen, 1992). Using Fisher’s z-transform of correlations ($r_\text {z} = 0.21, SD_\text {z} = 0.22$) and assuming the transformed values to be normally distributed, we ran a simulation 500 times. We found that, with $N = 20$, obtaining 24 trials per participant for a single transition condition (e.g., REG5-RAND5, after excluding trials with gaps) is sufficient to achieve a statistical power of $1-\beta = 0.89$. Similarly, collecting 48 trials per participant for two transition conditions (REG5-RAND5 and REG5a-REG5b together, after excluding trials with gaps) is sufficient to reach $1-\beta = 0.96$ with $\alpha = 0.05$ for detecting correlations across modalities.

Twenty participants attended the experiment (13 female, M_age_: 24.4, SD_age_: 7.51, mean M_logMAR_: -0.1, SD_logMAR_: 0.15). Participants were mostly university students and were compensated with either course credit or 12.5 EUR per hour. The experiment took approximately 2 hours.

#### Materials

Participants observed a set of 120 trials (30 REG5-RAND5, 30 REG5a-REG5b, and 60 REG5) for each modality. In contrast to our earlier experiments and previous studies (Zhao et al., 2019; Basgol et al., 2025), in which participants were presented with randomly generated, different sets of trials, all participants in this experiment received the same set of trials that were generated before the data collection (but in individually-randomised orders).

The frequency pool included nine pre-selected tones: three base frequencies (222, 292, and 384 Hz) and their two successive octaves (444, 584, 768 Hz and 888, 1168, 1536 Hz). Similarly, the position pool included the positions created by a 3 x 3 grid (without grey-coloured reference locations to keep presentations conceptually similar; see Fig. 1d). Unbeknownst to the participants, these pools were mapped: frequencies and dot positions were associated. High tones (low tones) were associated with higher (lower) locations in the grid to arrange for the visual and auditory patterns to be represented as similarly as possible (see the spatial-musical association of response codes, the SMARC effect, Rusconi et al., 2006; see Fig. 1d). When sampling sequences, we ensured that each visual sequence had an isomorphic auditory counterpart by choosing frequencies according to this predefined mapping (a standard practice in the statistical learning literature; Conway & Christiansen, 2005). This procedure created isomorphic visual and auditory sequences.

To ensure sufficient variability for correlational analyses, transitions involving differences of 3, 4, and 5 items (i.e., frequencies and dot locations) between patterns (first 5 items before and after transitions) were equally represented. Trials with zero circular Hamming distance (i.e., identical patterns differing only by a phase shift) were excluded, as such transitions could be imperceptible under rapid and continuous presentation (these trials were not excluded in Experiments 1 and 2).

Participants began the experiment with a block from one modality (e.g., auditory). Then they continued with a block from the other modality (e.g., visual), with the trial order randomised across participants. Notably, each pair of successive blocks, namely 1-2, 3-4, 5-6, included the identical sequences presented in different modalities in the same order. We kept blocks close in time given that pupil response systematically varies as a function of time (Drew et al., 2023).

Due to the long block duration (approximately 7 minutes), participants were unlikely to discover these associations; indeed, none of the participants reported being aware of it. The order of sequences was randomised for each participant to prevent time-varying factors (such as fatigue and warm-up effects) from being associated with specific sequences (Sirois & Brisson, 2014; Winn et al., 2018; Drew et al., 2023).

The length of trials was 7.5 s, and transitions occurred between 2.5 and 3.5 s after the sequence onset with a granularity of 0.25 s. REG5 sequences consisted of 30 repetitions (150 items), and REG5s in REG5-RAND5 and REG5-REG5b were violated after 10-14 repetitions (50-70 items). We shortened the trial duration relative to Experiments 1 and 2 to keep the experiment below 2 hours and reduce the risk of pupil fatigue (Winn et al., 2018; Drew et al., 2023).

Participants were instructed to detect gaps occurring between 0.5 s after sequence onset and 1 second before sequence offset, which occurred with a probability of 20%. Gaps in auditory sequences were set to 0.2 s, whereas in visual sequences, they were set to 0.4 s.

#### Procedure

Participants were shown examples of auditory and visual sequences (with gaps) and began the practice session with their assigned modality (for example, auditory); then, they continued the practice block for the other modality (for example, visual). Similar to the practice session, they began the main experiment with their assigned modality (e.g., auditory) and then continued with the alternate modality (e.g., visual). They completed a brief questionnaire, which now included a question about their perceived mental effort for the visual and auditory gap detection tasks. After the questionnaire, participants first rated the similarity between items and then assessed the saliency of transitions. Analyses and discussions associated with these measurements can be found in Supplementary Section S9.

Auditory stimuli were presented through headphones (diotically using Beyerdynamic DT-770 M 80 Ohm) connected to the display. All remaining procedural aspects were consistent with previous experiments.

### Analysis

#### Pupil responses

Compared with Experiments 1 and 2, we made a minor, preregistered adjustment to the pipeline of pupil size analysis. The analysis now focuses on the four-second (previously three-second) window following the transition, as 80% of pupil peaks occurred within this time frame in Experiments 1 and 2. Dummy transitions were kept consistent across isomorphic trials to ensure comparability. During preprocessing, 32 trials (approximately 1%) were excluded. Due to issues with the experimental setup, the left pupil were measured for two participants.

#### Time-series of pupil sizes

We compared time-series of pupil sizes across modalities using BF_01_ (which estimates the degree of evidence supporting the null hypothesis).

#### Correlations of pupil responses

We calculated scalar values for isomorphic transition pairs. Measurements include peak pupil size, the time of peak pupil response, mean pupil size and the minimum pupil size. We then computed correlations between these scalar values as follows:

- As detailed in the preregistration, we first examined whether participants showed comparable scalar pupil measures for isomorphic pairs. We calculated a Pearson correlation coefficient for each participant separately and tested if these correlations significantly differed from zero using a one-tailed *t*-test (following Fisher’s z-transformation).
- In an exploratory analysis, we examined whether scalar pupil measures were similar across modalities at the group level. We computed the average scalar value for each isomorphic transition and then assessed correlations between these averages. This reduced measurement noise, yielding more stable estimates.

### Results

#### Behavioural performance

**Fig. 4:**
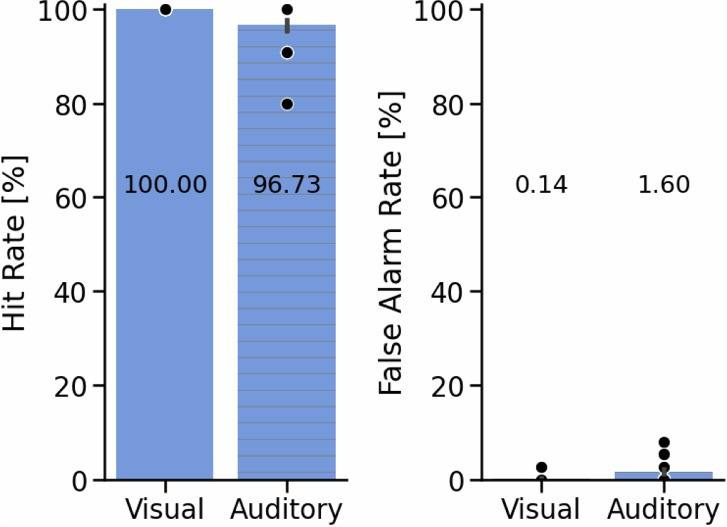
Gap detection performances for REG5 in Experiment 3. Error bars indicate between-participant SEMs

Hit and false alarm rates were slightly different across modalities (see Fig. 4), with lower hit rates for audition than vision (REG5: Auditory = 0.967, Visual = 1.00, $BF_{10}$ = 3.25, *p* = .017, 95% CI = [-0.000590, -0.0000645], $d_z$ = -0.598; for the average of all conditions: Auditory = 0.927, Visual = 0.988, $BF_{10}$ = 9.19, *p* = .005, 95% CI = [-0.001, -0.000208], $d_z$ = -0.728) and higher false alarm rates (REG5: Auditory = 0.016, Visual = 0.0014, $BF_{10}$ = 3.29, *p* = .017, 95% CI = [0.0000293, 0.000264], $d_z$ = 0.6; for the average of all conditions: Auditory = 0.015, Visual = 0.010, $BF_{10}$ = 0.265, *p* = .592, 95% CI = [-0.000116, 0.000197], $d_z$ = 0.125). Note that the gap intervals were longer for vision.

We analysed additional performance variables. Participants’ reaction times (RTs) did not differ (Auditory = 958 ms, Visual = 884 ms, BF_10_ = 0.39, *p* = .290, 95% CI = [-69.58, 218.12], *d*_z_ = 0.32) and they did not find the auditory task harder than the visual task (BF_10_ = 0.371, *p* = .316, 95% CI = [-0.31, 0.91], *d*_z_ = 0.213). Their perception of effort was correlated (*r* = .590, BF_10_ = 9.119, *p* = .006, 95% CI = [0.2, 0.82]).

**Fig. 5:**
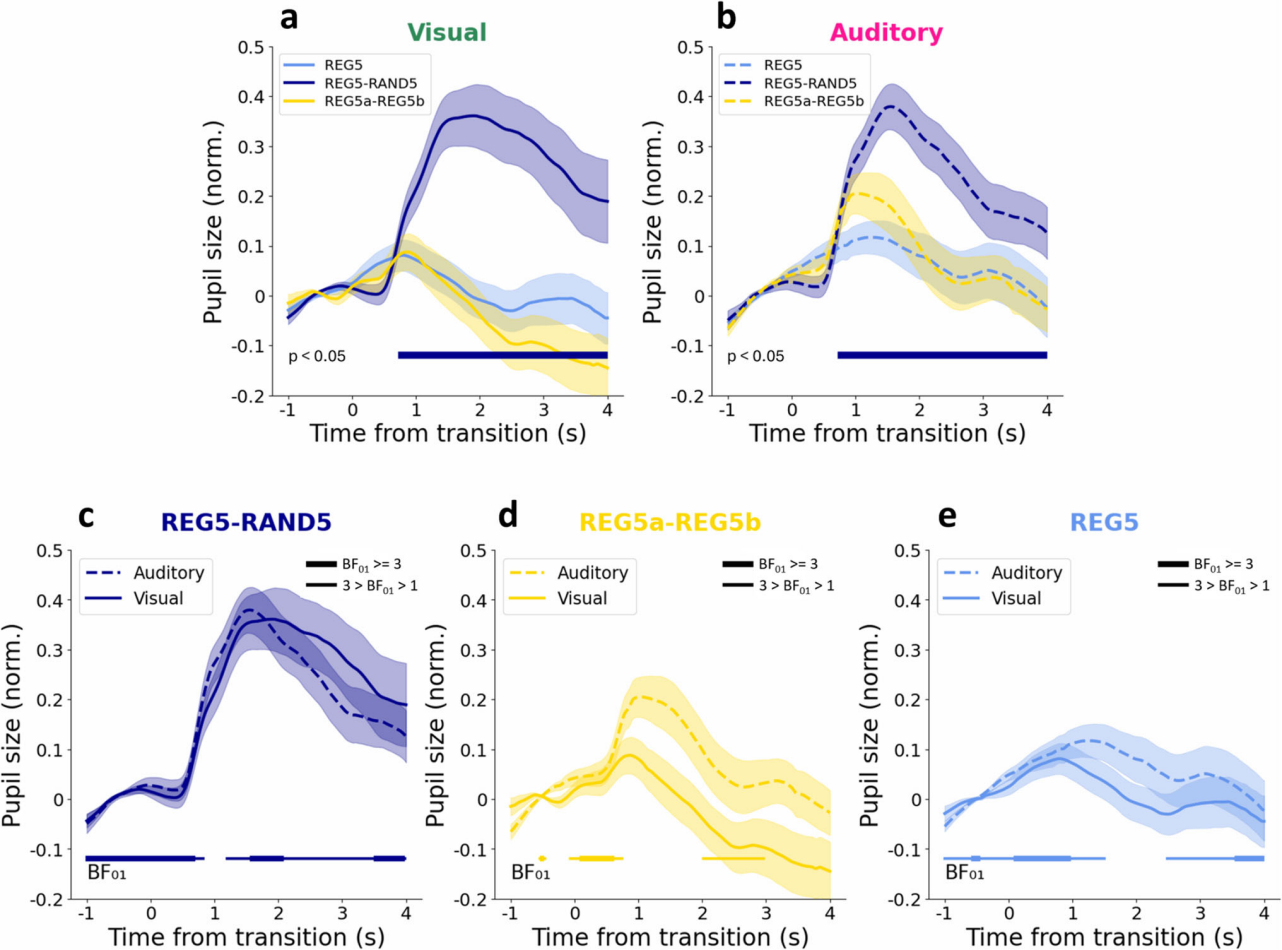
Time-series of pupil sizes across modalities in Experiment 3. Pupil responses in Experiment 3. Baseline-corrected pupil size for **a** visual and **b** auditory modalities. Similar to previous experiments, the REG5-RAND5 condition evoked pupil dilation responses. In contrast, the REG5a-REG5b condition did not trigger substantial pupil dilation. **c** The REG5-RAND5 condition led to similar pupil responses across modalities. **d** However, in the REG5a-REG5b condition, the auditory modality showed a slight increase compared to the visual modality, but this difference was not statistically significant. **e** The main pupil trend for the REG5 condition was similar across modalities, with the auditory modality showing slightly larger responses. Shaded areas indicate the between-participant SEMs. Coloured horizontal lines indicate regions where cluster-level statistics $p < .05$ in (a) and (b); and they indicate BF_01_ for (c), (d), and (e) to estimate the amount of evidence for the null hypothesis (suggesting no difference between the distributions). The thin line corresponds to BF_01_ > 1, and the thick line corresponds to BF_01_ > 3. We also calculated cluster-level statistics for (c), (d), and (e), which did not suggest a statistical difference (*p* > .05)

#### Time-series of pupil sizes

The REG5-RAND5 condition evoked remarkably similar pupil dilations across modalities (see Fig. 5a and b, and, for a comparison, see Fig. 5c). However, unlike Experiments 1 and 2, the REG5a-REG5b condition did not evoke strong pupil dilations (see Fig. 5a and b). A slight increase in pupil size was observed in the auditory modality compared to the visual modality, but the difference was not substantial (see Fig. 5d).

Cluster-based permutation statistics did not reveal a reliable difference between pupil responses across modalities. We then calculated BF_10_ for each time step, with none exceeding the threshold of 3. We then assessed the evidence for the null hypothesis using BF_01_ (see Fig. 5c, d, and e). Overall, pupil responses were similar across modalities with momentary deviations in REG5a-REG5b and REG5.

**Fig. 6:**
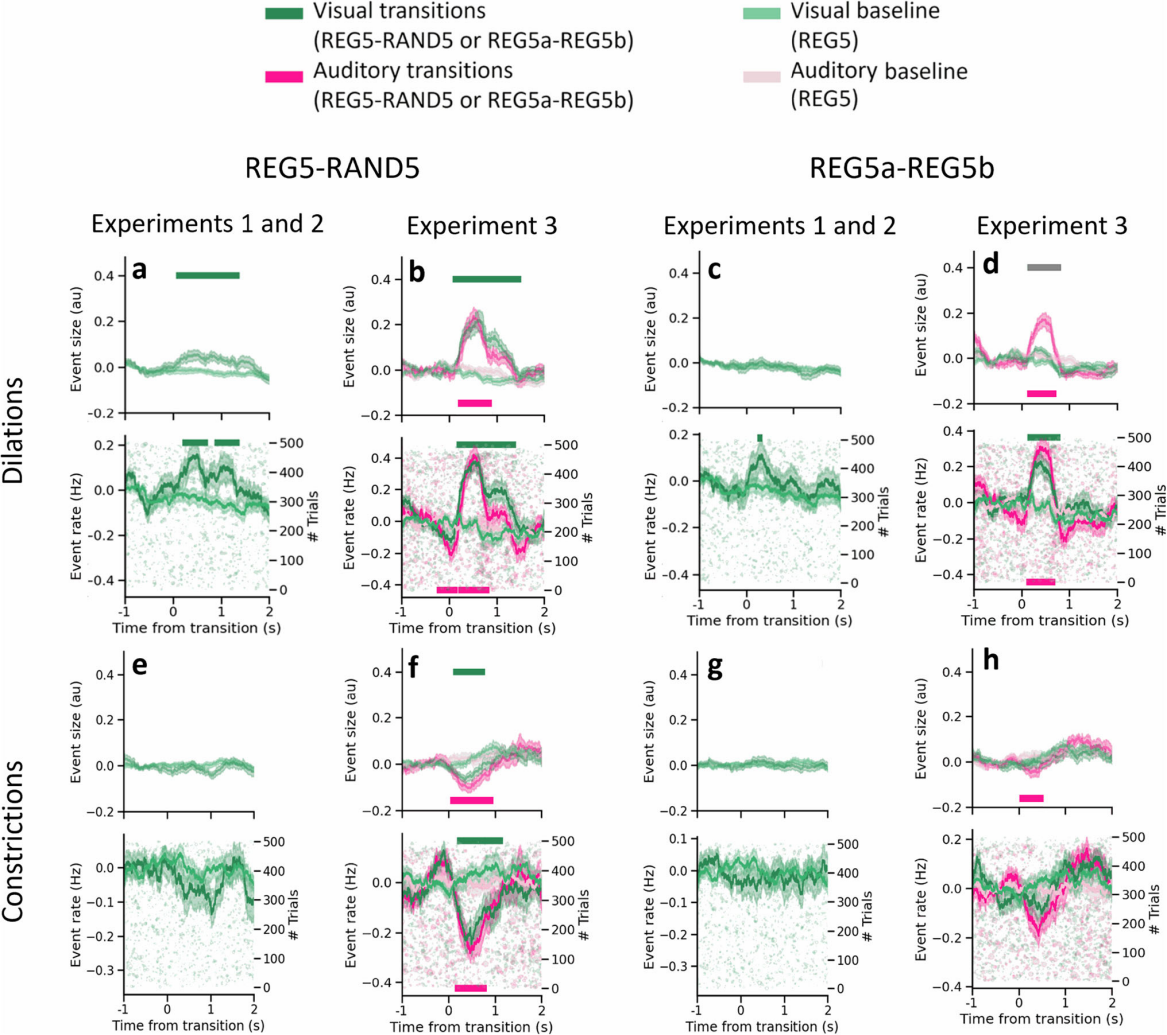
Pupil dilation events and their size across experiments. Baseline-corrected rates and sizes of dilation events. Note that **(a, b, c, d)** show dilation events; **(a, b)** for REG5-RAND5 and **(c, d)** for REG5a-REG5b conditions. **(e, f, g, h)** show constriction events; **(e, f)** for REG5-RAND5 and **(g, h)** for REG5a-REG5b conditions. The upper figures show the sizes of events, whereas the lower figures show event rates, regardless of the magnitude of the event. **(a, b)** Pupil dilation event rates and magnitudes are consistent in REG5-RAND5 across experiments. **(c, d)** These figures are also similar in REG5a-REG5b;. However, they are not as consistent as in REG5-RAND5 (compare Experiment 3 with Experiments 1 and 2). **(e, f)** Only in Experiment 3 do we observe clear constriction events, mirroring the dilation rates. Modalities also appeared to behave differently across conditions in their effects on pupil size and rates. In Experiment 3, **(b)** dilation rates and magnitudes are similar for the REG5-RAND5 condition; whereas, **(d)** they diverge for the REG5a-REG5b condition, where auditory modality did lead to a pupil size increase; whereas visual modality did not. Experiments 1 and 2 agree that visual modality does not lead to a dilation event size increase for REG5a-REG5b but for REG5-RAND5 **(a, c)**. Coloured horizontal lines indicate regions where cluster-level statistics $p < .05$ for transition and baseline conditions. The grey horizontal line in **(d)** indicates the difference between modalities. There was no reliable difference between auditory and visual baseline conditions (REG5). Dots in pupil rate figures correspond to dilation and constriction events in transition conditions.

#### Pupil events and their magnitudes

We conducted an exploratory analysis based on pupil events. For both REG5-RAND5 and REG5a-REG5b, dilation event rates and amplitudes were consistent (see Fig. 6a and b) across experiments with visual sequences. However, the effect of the REG5a-REG5b condition on pupil size was weaker (compare Fig. 6c and d).

Pupil events showed similar results for the REG5-RAND5 conditions (see Fig. 6b for dilations and Fig. 6d for constrictions). However, although visual and auditory REG5-REG5b conditions were highly similar in terms of the number of dilation events, they were different in size: the auditory REG5a-REG5b resulted in larger dilation events than its visual counterpart (Fig. 6d).

This analysis complements pupil-size averaging: the absence of a pupil-size increase in the REG5a-REG5b condition does not necessarily imply that pupil responses to transitions are absent, and it suggests that there may be differences across modalities in how they affect pupil responses (compare Figs. 5d and 6d).

#### Correlations of pupil responses

**Fig. 7:**
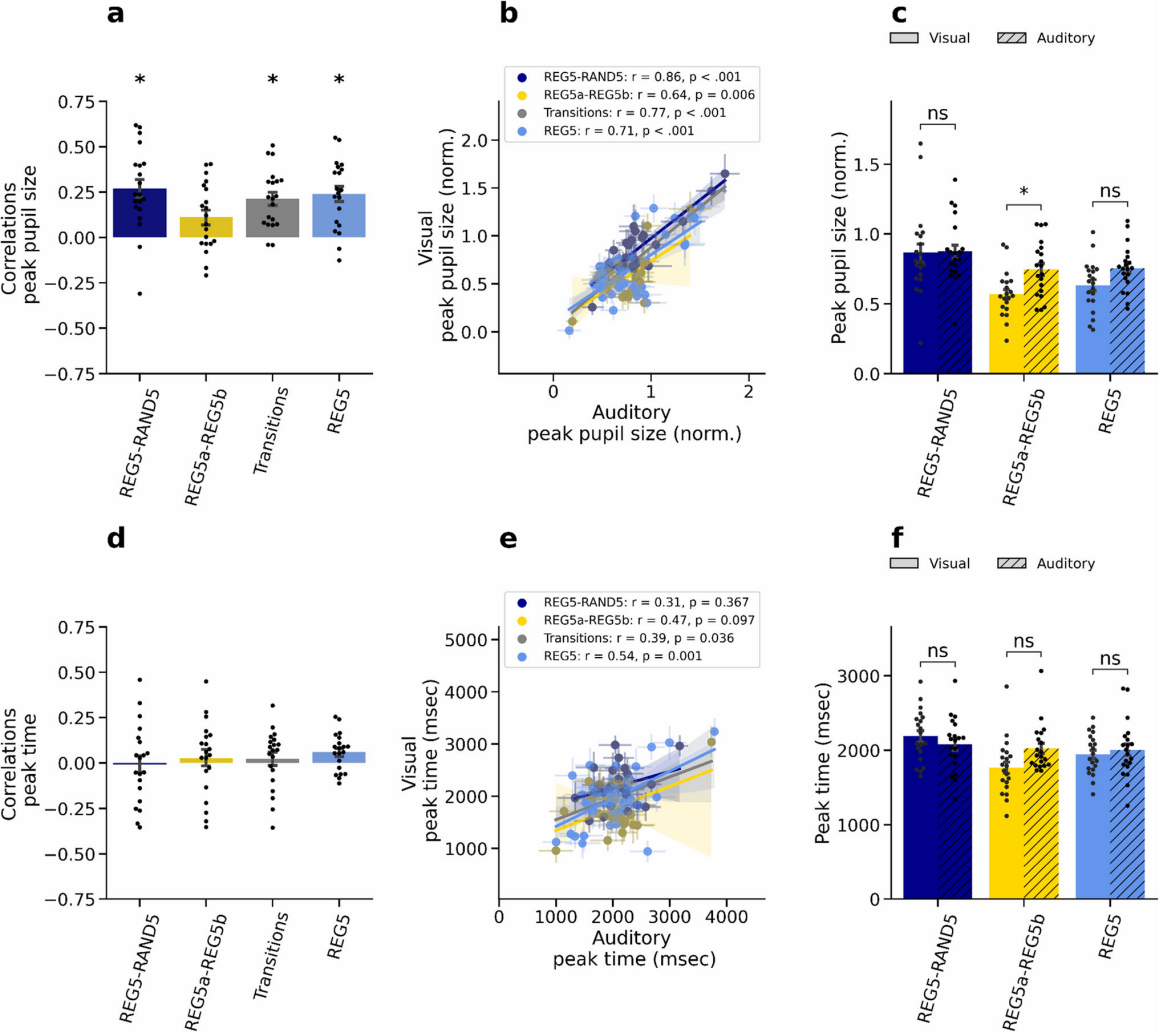
Comparisons of scalar values extracted from pupil traces in Experiment 3. The first row corresponds to peak pupil size, and the second corresponds to their time. Columns correspond to different types of comparisons: participants’ correlations, group correlations based on averages of isomorphic transitions, and differences of scalar values. Shaded areas and error bars indicate between-participant SEMs. Asterisks denote where *p*_corr_ $\boldsymbol{< 0.05}$

We conducted three analyses: We first estimated correlations within a participant (see Fig. 7a and Table 1). Second, we examined correlations at the group level by averaging scalar values (see Fig. 7b; Table 2). Finally, we compared scalar pupil measures across modalities (see Fig. 5). *p*-values reported in Tables 1, 2, and 3 were corrected on a per-table basis (using the Holm-Bonferroni method).

**Table 1:** Correlations of participants

	Statistics	*t*	*M*_r_	%95 CI	BF_10_	*p*_corr_	*d*
dv	Conditions						
Peak pupil size	**REG5-RAND5**	**5.06**	**0.28**	**[0.19, 1]**	**768.94**	$\boldsymbol{< .001}$	**1.13**
	REG5a-REG5b	2.69	0.12	[0.04, 1]	7.58	0.065	0.6
	**Transitions**	**5.73**	**0.22**	**[0.16, 1]**	$\boldsymbol{> 1000}$	$\boldsymbol{< .001}$	**1.28**
	**REG5**	**5.48**	**0.25**	**[0.17, 1]**	$\boldsymbol{> 1000}$	$\boldsymbol{< .001}$	**1.22**
Peak time	REG5-RAND5	-0.13	-0.01	[-0.09, 1]	0.47	0.812	0.03
	REG5a-REG5b	0.58	0.03	[-0.06, 1]	0.54	0.812	0.13
	Transitions	0.62	0.02	[-0.04, 1]	0.55	0.812	0.14
	REG5	2.55	0.06	[0.02, 1]	5.87	0.069	0.57
Mean size	**REG5-RAND5**	**4.34**	**0.22**	**[0.13, 1]**	**183.61**	**0.002**	**0.97**
	REG5a-REG5b	1.48	0.06	[-0.01, 1]	1.19	0.345	0.33
	**Transitions**	**4.67**	**0.15**	**[0.10, 1]**	**356.18**	**0.001**	**1.05**
	**REG5**	**4.5**	**0.18**	**[0.11, 1]**	**248.79**	**0.001**	**1.01**
Minimum size	REG5-RAND5	2.4	0.12	[0.03, 1]	4.54	0.081	0.54
	REG5a-REG5b	1.55	0.09	[-0.01, 1]	1.29	0.345	0.35
	Transitions	2.67	0.1	[0.03, 1]	7.25	0.065	0.6
	**REG5**	**3.26**	**0.13**	**[0.06, 1]**	**21.8**	**0.02**	**0.73**

Individual correlations based on isomorphic pairs of visual and auditory sequences were calculated for each participant. The correlations were then compared to 0 using a one-tailed *t*-test. $\textit{M}_r$ corresponds to the mean of individual correlations; 95% CI denotes the one-sided confidence interval. The statistical metrics BF_10_, *p*_corr_, and *d*_z_ refer, respectively, to the Bayes factor, the corrected *p*-values of these correlations, and effect sizes

**Table 2:** Correlations across Isomorphic Transitions

	Statistics	*r*	%95 CI	BF_10_	*p*_corr_
dv	Conditions				
Peak pupil size	**REG5-RAND5**	**0.86**	**[0.71, 0.94]**	$\boldsymbol{> 1000}$	$\boldsymbol{< .001}$
	**REG5a-REG5b**	**0.64**	**[0.32, 0.83]**	**56.96**	**0.006**
	**Transitions**	**0.77**	**[0.62, 0.86]**	$\boldsymbol{> 1000}$	$\boldsymbol{< .001}$
	**REG5**	**0.71**	**[0.53, 0.83]**	$\boldsymbol{> 1000}$	$\boldsymbol{< .001}$
Peak time	REG5-RAND5	0.31	[-0.11, 0.63]	0.71	0.367
	REG5a-REG5b	0.47	[0.09, 0.74]	3.33	0.097
	**Transitions**	**0.39**	**[0.12, 0.61]**	**7.08**	**0.036**
	**REG5**	**0.54**	**[0.31, 0.72]**	**393.55**	**0.001**
Mean size	**REG5-RAND5**	**0.8**	**[0.58, 0.91]**	$\boldsymbol{> 1000}$	$\boldsymbol{< .001}$
	REG5a-REG5b	0.35	[-0.06, 0.66]	0.96	0.367
	**Transitions**	**0.64**	**[0.44, 0.79]**	$\boldsymbol{> 1000}$	$\boldsymbol{< .001}$
	**REG5**	**0.53**	**[0.3, 0.71]**	**304.7**	**0.001**
Minimum size	**REG5-RAND5**	**0.64**	**[0.32, 0.83]**	**53.21**	**0.006**
	REG5a-REG5b	0.2	[-0.22, 0.56]	0.39	0.367
	**Transitions**	**0.46**	**[0.21, 0.66]**	**38.16**	**0.006**
	REG5	0.25	[-0.04, 0.5]	0.72	0.367

Correlations were calculated based on the mean of visual and auditory transitions, reflecting group-level responses. *r* denotes the correlation coefficient; 95% CI denotes the confidence interval. The statistical metrics BF_10_ and *p*_corr_ refer, respectively, to the Bayes factor and the corrected *p*-values of these correlations

##### Peak and mean pupil size

Peak pupil size was correlated (see Table 1, one-tailed *t*-tests were used as specified in the preregistration) for the majority of participants (see Fig. 7a). Note, however, that the REG5a-REG5b condition showed a weaker correlation (*M*_r_ = 0.12) than the REG5-RAND5 condition (*M*_r_ = 0.28; difference: *t* = 2.658, *BF*$_{10}$ = 3.556, *p* = 0.016, *d*_z_ = 0.763, 95% CI = [0.030, 0.280]). Group responses calculated for isomorphic transitions agreed with this observation (see Fig. 7b and Table 2), again the correlation for REG5a-REG5b was numerically weaker (*r* = 0.64) than REG5-RAND5 (*r* = 0.86; however, the difference was not statistically strong, *z* = 1.734, *p* = 0.083, 95% CI = [-0.070, 1.140]). Mean pupil size was consistent with peak pupil size (see Tables 1 and 2; difference: *z* = 2.376, *p* = 0.018, 95% CI = [0.128, 1.338] with a statistically strong estimate). The REG5a-REG5b condition did not lead to consistent correlations compared to the REG5-RAND5 condition, following peak pupil size (*t* = 2.3, *BF*$_{10}$ = 2.2, *p* = 0.028, *d*_z_ = 0.75, 95% CI = [0.020, 0.280]).

The REG5-RAND5 condition led to the same peak pupil magnitudes (see Fig. 7c and Table 3); This measure was slightly different for the REG5a-REG5b and REG5 conditions (see Table 3).

##### Peak time

The time of pupil peaks was not consistently correlated within participants (see Fig. 7d and Table 1); it became correlated when all transitions together were considered (see Fig. 7e and Table 2). The absence of a correlation in REG5-RAND5 may reflect that this condition induces sustained violations, to which the pupil can continue to respond. The time of peak pupil size was similar across conditions (note that *t*-tests were conducted after log transformation). Only the REG5a-REG5b condition appeared different, but this effect did not survive the multiple comparison correction (see Table 3).

##### Minimum pupil size

As an exploratory analysis, we examined minimum pupil size, as it may reflect a decrease in uncertainty (Milne et al., 2021). The REG5-RAND5 condition seemed to be correlated (see Table 1 for a weak correlation and Table 2 for a stronger correlation).

##### Control analysis

Correlations reflect structural similarities between isomorphic transitions. Still, it is reasonable to question if these correlations may have been modulated by other factors (despite our randomisation attempts). We re-ran these analyses to address this possible concern, determining "fake" isomorphic pairs. These fake pairs were determined as the closest trials (in terms of time) to the original pair within the same condition (and block); thereby assessing whether correlations are due to the dynamics of the experiment. Out of 32 tests estimating correlations, only one test yielded a BF_10_ greater than 3, which is the correlation of modalities for REG5 in terms of peak size ($M_\textit{r}$ = 0.09, BF_10_ = 12.59, *p*_corr_ = 0.063). No other comparison yielded a BF_10_ > 3 (with all other corrected *p*-values > .476).

##### Correlations of pupil events

We conducted similar analyses based on dilation events. While the number of dilation events did not correlate across modalities (note that the maximum number of events in a trial was only 4), the total magnitude of events showed strong correlations (but not in REG5a-REG5b; see Supplementary Figure S5).

**Table 3:** Pupil differences across modalities

	Statistics	*t*	*M*_A_	*SEM*_A_	*M*_V_	*SEM*_V_	95% CI	BF_10_	*p*_corr_	$d_z$
dv	Conditions									
Peak pupil size	REG5-RAND5	0.22	0.88	0.05	0.87	0.07	[-0.09, 0.11]	0.24	1.0	0.04
	**REG5a-REG5b**	**3.71**	**0.75**	**0.04**	**0.57**	**0.04**	**[0.08, 0.28]**	**25.76**	**0.018**	**0.98**
	REG5	3.1	0.75	0.04	0.63	0.04	[0.04, 0.2]	8.05	0.064	0.71
Peak time	REG5-RAND5	-1.18	2080.0	78.0	2191.0	76.0	[-0.15, 0.04]	0.43	1.0	0.32
	REG5a-REG5b	2.98	2025.0	69.0	1770.0	81.0	[0.04, 0.25]	6.38	0.077	0.82
	REG5	0.66	2006.0	83.0	1946.0	59.0	[-0.05, 0.09]	0.28	1.0	0.13
Mean size	REG5-RAND5	-0.73	0.21	0.04	0.24	0.05	[-0.09, 0.04]	0.3	1.0	0.12
	REG5a-REG5b	2.07	0.08	0.04	-0.03	0.04	[-0.0, 0.23]	1.35	0.415	0.62
	REG5	1.43	0.07	0.04	0.01	0.04	[-0.02, 0.13]	0.56	1.0	0.3
Minimum size	REG5-RAND5	-2.46	-0.58	0.04	-0.47	0.04	[-0.21, -0.02]	2.51	0.214	0.6
	REG5a-REG5b	0.01	-0.67	0.05	-0.67	0.05	[-0.13, 0.13]	0.23	1.0	0.0
	REG5	-1.53	-0.67	0.05	-0.6	0.04	[-0.17, 0.03]	0.63	0.998	0.32

Differences of scalar pupil values extracted from pupil traces. *t* denotes the value of *t*-test, *M*_A_ and *M*_V_ correspond to the mean of scalar pupil values; *SEM*_A_ and *SEM*_V_ are corresponding standard error of the means (SEMs). 95% CI denotes confidence intervals. Statistical metrics, BF_10_ and *p*_corr_, refer, respectively, to the Bayes factor and the corrected *p*-values of these correlations. $d_z$ corresponds to effect sizes

## General discussion

Given the sensitivity of pupil responses to prediction errors (Basgol et al., 2025; Zhao et al., 2019), and building on prior evidence indicating cross-modal similarities in the processing of deviant stimuli (Grundei et al., 2023; Sabio-Albert et al., 2025; Planton & Dehaene, 2021), we adapted the rapid tone presentation paradigm used in audition to vision (Barascud et al., 2016; Basgol et al., 2025; Southwell et al., 2017; Southwell & Chait, 2018; Zhao et al., 2019) and investigated pupil dynamics to changes within visual and auditory sequences.

### Regularity violations and pupil dilation responses

Our main results in the visual modality were consistent with previous auditory observations (Basgol et al., 2025; Zhao et al., 2019). The emergence of regularities (RAND5-REG5, Experiment 1) does not lead to pupil dilations, whereas violations of regularities by random patterns (REG5-RAND5; Experiments 1, 2, and 3) and by regular patterns does (REG5a-REG5b; Experiments 1, 2, see Figure 6 and S5). Therefore, our results support the conclusion that pupil dilations are associated with domain-general reset of internal models in response to abrupt violations of predictive relationships (i.e., unexpected uncertainties triggering the LC-NE system activity;  Basgol et al., 2025; Zhao et al., 2019). However, there were two empirical irregularities: the magnitude of pupil dilation to REG5a-REG5b transitions was consistently smaller than REG5-RAND5 (compared to the previous study, Supplementary Section S4) and its magnitude was inconsistent across experiments; see Figs. 2b,  3b, and  5). We return to this issue after presenting the remaining empirical results.

### Pupil size: cross-modal similarities and differences

The REG5-RAND5 conditions elicited remarkably similar pupil dilations (see Experiment 3, Fig. 5c). This resemblance is evident not only in scalar measures but also in the overall pupil dynamics. A comparable, albeit weaker, similarity was observed between the REG5a-REG5b conditions across modalities.

As an exploratory analysis, we compared pupil responses across modalities without transitions (i.e., REG5). Interestingly, pupil responses are still correlated. This suggests that pupil size may reflect not only changes due to transitions, b ut also inferences of participants in response to REG5 sequences that could be interpreted as the general level of uncertainty (Milne et al., 2021), which has been shown to lead to tonic increases in pupil size (Gesztesi & Pajkossy, 2025; Filipowicz et al., 2020; Nassar et al., 2012; Pajkossy et al., 2023).

Crucially, we based cross-modal correlations on isomorphic transitions rather than participant-wise means, as the latter can be confounded by individual differences in pupil responsiveness. By analysing responses per transition (either for a participant or all participants as a group), we effectively eliminated participant-specific biases.

### Pupil events: cross-modal similarities and differences

We extracted pupil events putatively associated with the LC-NE system activity (Basgol et al., 2025; Joshi et al., 2016; Megemont et al., 2022; Milne et al., 2021; Zhao et al., 2019; Reimer et al., 2016). Transitions in REG5-RAND5 showed a consistent increase (see Fig. 6a and 6b; in line with previous studies, Basgol et al., 2025); on the other hand, transitions in REG5a-REG5b showed a strong increase in Experiment 3 (see Fig. 6d), in which sequences were identical across participants and thus reduced pupil-response variability, whereas it showed smaller increases in Experiments 1 and 2 (see Fig. 6c).

Dilation events and their magnitudes increased after transitions in REG5-RAND5 conditions (see Fig. 6) with a remarkably similar degree across modalities. In contrast, the auditory and visual REG5a-REG5b conditions differed in terms of the size of dilation events (but not in rates; see Fig. 6d; and see Fig. 6c for the events in visual sequences), consistent with the numerically larger pupil responses in the auditory modality (Fig. 5d; see also Klingner et al., 2011).

### Integrating the findings

#### Domain-general responses with modality constraints

Structured learning of patterns occurs across perceptual domains. Yet, the underlying mechanisms vary by modality and the presentation mode, suggesting modality-specific systems (Conway, 2020; Conway & Christiansen, 2009; Frost et al., 2015).

Strong cross-modal correlations, especially in REG5-RAND5, support (but do not prove) the idea of a domain-general response to prediction errors (Grundei et al., 2023; Planton & Dehaene, 2021; Sabio-Albert et al., 2025) that seems to trigger activity of the LC-NE system and informs the arousal system about the moments of model reset. Saliency reports of participants, interpreted as an evaluation of changing uncertainties upon transitions, suggest a similar conclusion (see Supplementary Section S9).

On the other hand, divergences in REG5a-REG5b across modalities (albeit with correlated responses) suggest modality-specific differences (see Figs. 6d, 7c, and f). In the following section, we discuss differences between the REG5-RAND5 and REG5a-REG5b conditions.

#### Differences between regularity violations

Our data included two findings about the REG5a-REG5b transitions that need further scrutiny. First, the magnitude of dilation associated with these was consistently smaller than for REG5-RAND5. Second, this magnitude was not consistent across experiments.

The comparison between REG5a-REG5b and REG5-RAND5 provides an important window onto the effects on pupil dilation of evolving certainty (REG5b) versus persistent uncertainty (RAND5). From a statistical viewpoint, both these transitions are initially associated with unexpected uncertainty. The resulting transient uncertainty, which is expected after an unexpected change, and the associated violations of predictions both rapidly reduce in REG5b, but persist in RAND5 (evident also in our quantitative predictions of surprise in Fig. 2d). We argue that it is this difference that determines the observed dilation, at least when filtered by the low-pass pupil response function (Hoeks & Levelt, 1993; Basgol et al., 2025). This reasoning leads one to expect that REG5a-REG5b will evoke less pupil dilation than REG5-RAND5 (and, presumably, also less than REG10-RAND10, which was one of the conditions reported in Basgol et al., 2025).

Nevertheless, the processes underpinning the persistent elevation following the switch to RAND5 in REG5-RAND5 are not yet fully understood. One possibility is that pupil dilation simply follows surprise (Fig. 2d). This surprise should itself become predictable (i.e., become a form of expected uncertainty), which Yu and Dayan (2005) suggested would be represented by ACh. This neuromodulator could itself impact tonic pupil dilation (Milne et al., 2021; Lloyd et al., 2023; Reimer et al., 2016; Nelson & Mooney, 2016; Mridha et al., 2021; Yu & Dayan, 2005). However, expected uncertainty includes both reducible (epistemic) and irreducible (aleatoric) components. The REG5a-REG5b transition illustrates the former; one might expect the latter ultimately to be filtered out as a form of adaptation. It would be interesting to design an experiment to test this cleanly.

There are two inconsistencies across experiments in the dilation to REG5a-REG5b. In Experiments 1 and 2, the transition evoked significant excess dilation (Fig. 2b and c; Fig. 3b and c), despite no considerable difference in the size of dilation or constriction events (Fig. 6c and g). In the visual condition of Experiment 3, there was no considerable dilation (Fig. 5a and d), but there were excess dilation events (Fig. 6d and h). In the auditory condition of Experiment 3, there was also no considerable dilation (Fig. 5b and d), but there were excess dilation events that were also larger (Fig. 6d and h). These observations imply that the signal-to-noise ratio for these transitions may be low, arising inconsistent and weak dilations. However, it is also possible that the visual regularities are harder to learn than the auditory ones; consequently, both the violation and establishment of the statistical structure are weaker, leading to smaller signals (Conway, 2020). The structure of the regularities in REG5b in Experiment 3 was also slightly different from that in Experiments 1 and 2, which may have further influenced the results.

While the current account emphasises uncertainties, it would be unsafe to assume that the pupil exclusively reflects these processes. Pupil size is sensitive to many changes in internal states (Rylan et al., 2018; Strauch et al., 2022; Grujic et al., 2024). For example, pupil-linked changes have been related to shifts in arousal, exploration-exploitation trade-off, effort, stress, circadian phase and metabolic or motivational state (Grujic et al., 2024). Therefore, the pupil provides a composite read-out of several partly overlapping control systems rather than a pure index of any single computational variable. Nonetheless, in the present experiments the close temporal locking of pupil responses to sequence transitions makes it plausible that unexpected uncertainty-related signals constitute the main contributors to the effects we report, while fluctuations in internal variables may modulate their expression and thereby contribute to the variability observed, for example, in responses to REG5a-REG5b transitions.

### Limitations and further research

The reduced and unstable REG5a-REG5b response, together with the weaker post-phasic reduction in the REG5-RAND5 condition relative to earlier work, leaves some important questions. Future studies should first manipulate expected uncertainty independently of transition type, for example by varying the conditional variability of the pre-transition sequence while keeping the REG5-RAND5 and REG5a-REG5b transitions fixed. We predict that higher expected uncertainty will attenuate phasic dilations, particularly for REG5a-REG5b. A second possibility is to examine how learning speed and pattern familiarity modulate responses to transitions between regularities, by comparing REG5a-REG5b transitions with novel versus pre-familiarised post-transition patterns. If novel REG5b patterns evoke larger and more prolonged phasic dilations than familiar patterns (or increase in event rates), this would support the idea that rapid inference of the new regularity shortens the period of elevated unexpected uncertainty and thus explains the weaker, less stable REG5a-REG5b effects at the group level.

Minor dilation events and global pupil size do not covary in a simple, one-to-one manner. The presented dissociations indicate that the presence and strength of event-level responses can diverge from the shape and magnitude of the global pupil trace, and that a weak or absent sustained mean increase for REG5a-REG5b does not necessarily imply the absence of phasic, uncertainty-related responses. To clarify these relationships, future work can model the pupil trace as the output of a generative process in which dilation events (Joshi et al., 2016; Reimer et al., 2016; Megemont et al., 2022) are driven by computational variables such as unexpected uncertainty (Basgol et al., 2025; Dayan & Yu, 2006; Yu & Dayan, 2005). These events then can be convolved with a pupil response function (Hoeks & Levelt, 1993; Basgol et al., 2025) with possible addition of tonic state variables associated with expected uncertainty and/or belief uncertainty (Nassar et al., 2012; Milne et al., 2021; Filipowicz et al., 2020). This approach would link pupil responses to sequence structure, and thereby help to interpret discrepancies such as the weaker and less consistent REG5a-REG5b effects across experiments. Such a model could also be used to investigate individual sensitivities to modalities.

In our visual paradigm, regularities were formed by spatio-temporal relationships between the positions of the white dot. Similar paradigms have been used before (Conway & Christiansen, 2005, 2009; Simon et al., 2016), where it has been found that participants extracted visual patterns better when stimuli were presented spatially compared to, for example, when they were presented as successive coloured patches (Conway & Christiansen, 2009; Hans et al., 1966; Robinson & Sloutsky, 2007). Therefore, a key question is how current results vary across different presentations within a modality (e.g., by varying durations of items or their complexities) and, if so, where any differences originate. For example, in the current paradigm, small changes in dot location after transitions may not significantly affect pupil size, not because of visual limitations, but due to the way the visual system functions. Vision, for example, is scale- and translation-invariant, which may render specific transitions to be easily integrated with prior information. One compelling experimental idea is to identify conditions in which the visual modality surpasses the auditory modality, thereby reversing the current pattern of results.

There is growing evidence that the visual system exhibits anisotropy, and the pupil is no exception. For instance, the pupillary light reflex is influenced differently by luminance from the top versus the bottom of a display (Cai et al., 2024). Some of the variability in pupil responses to visual regularity violations could be attributed to this effect.

Retinal illumination and covert attention to the illuminated portion of the screen lead to pupil constrictions (Binda et al., 2013; Mathôt et al., 2013; Strauch, 2024); therefore, white dots that do not appear in a predicted position might lead to small dilations (if the retina is on that spot). These studies used large and wide stimuli (usually occupying half of the screen), remaining on the screen for extended periods (in the order of seconds). In contrast, in our presentations, the size of white dots was small (maximum of 1° of visual angle), the dots were presented in a confined space (5° wide of visual angle) and presented quickly (20 Hz, in the order of ms). A natural extension would be to use a black dot to diminish this possible effect or investigate how dots with different luminance profiles affect pupil responses (Pan et al., 2022, 2024). Given the strong cross-modal consistency and applied gaze-related controls, any remaining effects of oculomotor behaviours are likely minimal. Indeed, saccade, microsaccade, and blink rates showed no modulation by transitions (see Supplementary Section S10).

Previous research has investigated the oddball effect on pupil size, for example, by simultaneously presenting auditory and visual stimuli (Liao et al., 2016b). Further research can explore how pupil size dynamically evolves within sequences when a new modality is introduced and how multimodal predictions are formed (Van Der Stoep et al., 2021; Rigato et al., 2016; Liu et al., 2024).

## Conclusion

Previous studies point to the possibility that the processing of regularities is modality-specific (Sherman et al., 2020; Conway, 2020; Barascud et al., 2016; Paavilainen, 2013; Frost et al., 2015; Canale, 2022), even if violations of those regularities may be detected through a domain-general mechanism (Sabio-Albert et al., 2025; Grundei et al., 2023; Planton & Dehaene, 2021). We observed that pupil dilation responses, often linked to such violations (Basgol et al., 2025; Nassar et al., 2012; Zhao et al., 2019), exhibit notable similarities across modalities. While these responses are correlated across domains, transitions with random patterns elicit more consistent effects than those with regular patterns. These findings highlight pupil dilation responses as a sensitive, modality-general index of the violation of statistical structure, while also revealing modality-specific differences in their processing.

## Supplementary Information

Below is the link to the electronic supplementary material.

Supplementary file 1 (pdf 4083 KB)

## Data Availability

The raw and processed data as well as materials associated with the experiment is publicly available on Zenodo (10.5281/zenodo.18613650).

## Code Availability

The associated code is publicly available on Zenodo (10.5281/zenodo.18613650).

## References

[CR1] Zekveld, A. A., Koelewijn, T., & Kramer, S. E. (2018). The pupil dilation response to auditory stimuli: Current state of knowledge. *Trends in Hearing,**22*, 2331–2165. 10.1177/2331216518777174 10.1177/2331216518777174 PMC6156203 30249172

[CR2] Gramfort, A., et al. (2013). MEG and EEG data analysis with MNE-Python. *Frontiers in Neuroinformatics,**7*, 267. 10.3389/fnins.2013.00267 PMC3872725 24431986

[CR3] Filipowicz, A. L., Glaze, C. M., Kable, J. W., & Gold, J. I. (2020). Pupil diameter encodes the idiosyncratic, cognitive complexity of belief updating. *elife,**9*, e57872. 10.7554/eLife.57872 10.7554/eLife.57872 PMC7289603 32420866

[CR4] Marois, A., & Vachon, F. (2018). Can pupillometry index auditory attentional capture in contexts of active visual processing? *Journal of Cognitive Psychology,**30*(4), 484–502.

[CR5] Milne, A. E., et al. (2021). Sustained pupil responses are modulated by predictability of auditory sequences. *The Journal of Neuroscience,**41*(28), 6116–6127. 10.1523/JNEUROSCI.2879-20.2021 34083259 10.1523/JNEUROSCI.2879-20.2021 PMC8276747

[CR6] Soltani, A., & Izquierdo, A. (2019). Adaptive learning under expected and unexpected uncertainty. *Nature Reviews Neuroscience,**20*(10), 635–644. 10.1038/s41583-019-0180-y 10.1038/s41583-019-0180-y PMC6752962 31147631

[CR7] Nelson, A., & Mooney, R. (2016). The basal forebrain and motor cortex provide convergent yet distinct movement-related inputs to the auditory cortex. *Neuron*, *90*(3), 635–648. 10.1016/j.neuron.2016.03.031, https://www.sciencedirect.com/science/article/pii/S0896627316300228 10.1016/j.neuron.2016.03.031 PMC4866808 27112494

[CR8] Alamia, A., & Zénon, A. (2016). Statistical regularities attract attention when task-relevant. *Frontiers in Human Neuroscience,**10*, 42. 26903846 10.3389/fnhum.2016.00042 PMC4746264

[CR9] Alamia, A., et al. (2019). Pupil-linked arousal responds to unconscious surprisal. *The Journal of Neuroscience,**39*(27), 5369–5376. 10.1523/JNEUROSCI.3010-18.2019 31061089 10.1523/JNEUROSCI.3010-18.2019 PMC6607748

[CR10] Yu, A. J. (2012). Change is in the eye of the beholder. *Nature Neuroscience,**15*(7), 933–935. 10.1038/nn.3150 22735513 10.1038/nn.3150

[CR11] Yu, A. J., & Dayan, P. (2005). Uncertainty, neuromodulation, and attention. *Neuron*, *46*(4), 681–692. 10.1016/j.neuron.2005.04.026, https://linkinghub.elsevier.com/retrieve/pii/S0896627305003624 10.1016/j.neuron.2005.04.026 15944135

[CR12] Bach, M. (2006). The freiburg visual acuity test-variability unchanged by post-hoc re-analysis. *Graefe’s Archive for Clinical and Experimental Ophthalmology,**245*, 965–971. 10.1007/s00417-006-0474-4 17219125

[CR13] Hoeks, B., & Levelt, W. J. M. (1993). Pupillary dilation as a measure of attention: a quantitative system analysis. *Behavior Research Methods, Instruments, & Computers,**25*(1), 16–26. 10.3758/BF03204445

[CR14] Lloyd, B., et al. (2023). Pupil size reflects activation of subcortical ascending arousal system nuclei during rest. *elife,**12*, e84822. 10.7554/eLife.84822 37367220 10.7554/eLife.84822 PMC10299825

[CR15] Ferguson, B., Franconeri, S.L., & Waxman, S.R. (2018). Very young infants learn abstract rules in the visual modality. *Plos ONE*, *13*(1). 10.1371/journal.pone.0190185. 10.1371/journal.pone.0190185 PMC5749756 29293554

[CR16] Sherman, B. E., Graves, K. N., & Turk-Browne, N. B. (2020). The prevalence and importance of statistical learning in human cognition and behavior. *Current Opinion in Behavioral Sciences*, *32*, 15–20. 10.1016/j.cobeha.2020.01.015, https://linkinghub.elsevier.com/retrieve/pii/S2352154620300152 10.1016/j.cobeha.2020.01.015 PMC7108790 32258249

[CR17] Strauch, C., et al. (2022). Pupillometry as an integrated readout of distinct attentional networks. *Trends in Neurosciences*, *45*(8), 635–647. 10.1016/j.tins.2022.05.003, https://linkinghub.elsevier.com/retrieve/pii/S0166223622000972. 10.1016/j.tins.2022.05.003 35662511

[CR18] Strauch, C. (2024). The forgotten wave of early pupillometry research. *Trends in Neurosciences,**47*(8), 571–572. 38942651 10.1016/j.tins.2024.06.002

[CR19] Robinson, C. W., & Sloutsky, V. M. (2007) Visual statistical learning: Getting some help from the auditory modality. In *Proceedings of the Annual Meeting of the Cognitive Science Society*, (Vol. 29. p. 29).

[CR20] Conway, C. M., & Christiansen, M. H. (2005). Modality-constrained statistical learning of tactile, visual, and auditory sequences. *Journal of Experimental Psychology: Learning, Memory, and Cognition,**31*(1), 24–39. 10.1037/0278-7393.31.1.24 15641902 10.1037/0278-7393.31.1.24

[CR21] Conway, C. M., & Christiansen, M. H. (2009). Seeing and hearing in space and time: Effects of modality and presentation rate on implicit statistical learning. *European Journal of Cognitive Psychology,**21*(4), 561–580. 10.1080/09541440802097951

[CR22] Conway, C. M. (2020). How does the brain learn environmental structure? Ten core principles for understanding the neurocognitive mechanisms of statistical learning. *Neuroscience & Biobehavioral Reviews*, *112*, 279–299. 10.1016/j.neubiorev.2020.01.032, https://linkinghub.elsevier.com/retrieve/pii/S0149763419307122 10.1016/j.neubiorev.2020.01.032 PMC7211144 32018038

[CR23] Privitera, C. M., et al. (2010). Pupil dilation during visual target detection. *Journal of Vision,**10*(10), 3. 10.1167/10.10.3 20884468 10.1167/10.10.3

[CR24] Cohen, J. (1992). A power primer. *Psychological Bulletin,**112*, 155–159. 19565683 10.1037//0033-2909.112.1.155

[CR25] Duncan, C. C., et al. (2009). Event-related potentials in clinical research: Guidelines for eliciting, recording, and quantifying mismatch negativity, P300, and N400. *Clinical Neurophysiology*, *120*(11), 1883–1908. 10.1016/j.clinph.2009.07.045, https://www.sciencedirect.com/science/article/pii/S1388245709005185 10.1016/j.clinph.2009.07.045 19796989

[CR26] Funder, D. C., & Ozer, D. J. (2019). Evaluating effect size in psychological research: Sense and nonsense. *Advances in Methods and Practices in Psychological Science,**2*(2), 156–168. 10.1177/2515245919847202

[CR27] Prete, D. A., et al. (2022). The sound of silence: Predictive error responses to unexpected sound omission in adults. *European Journal of Neuroscience,**55*(8), 1972–1985. 10.1111/ejn.15660 35357048 10.1111/ejn.15660

[CR28] McLaughlin, D. J., et al. (2023). Give me a break! Unavoidable fatigue effects in cognitive pupillometry. *Psychophysiology,**60*(7), e14256. 10.1111/psyp.14256 36734299 10.1111/psyp.14256 PMC11161670

[CR29] Larson, E., et al. (2022). MNE-Python (1.2. 3) [computer software].

[CR30] Rusconi, E., et al. (2006). Spatial representation of pitch height: the SMARC effect. *Cognition*, *99*(2), 113–129. 10.1016/j.cognition.2005.01.004, https://linkinghub.elsevier.com/retrieve/pii/S0010027705000260. 10.1016/j.cognition.2005.01.004 15925355

[CR31] Sussman, E. S. (2005). Integration and segregation in auditory scene analysis. *The Journal of the Acoustical Society of America,**117*(3), 1285–1298. 10.1121/1.1854312 15807017 10.1121/1.1854312

[CR32] Maris, E., & Oostenveld, R. (2007). Nonparametric statistical testing of EEG and MEG data. *Journal of Neuroscience Methods,**164*(1), 177–190. 17517438 10.1016/j.jneumeth.2007.03.024

[CR33] Schröger, E., Marzecová, A., & SanMiguel, I. (2015). Attention and prediction in human audition: a lesson from cognitive psychophysiology. *European Journal of Neuroscience*, *41*(5). 10.1111/ejn.12816 PMC4402002 25728182

[CR34] Lieder, F., et al. (2013). Modelling trial-by-trial changes in the mismatch negativity. *PLOS Computational Biology,**9*(2), e1002911. 10.1371/journal.pcbi.1002911 23436989 10.1371/journal.pcbi.1002911 PMC3578779

[CR35] Lecaignard, F., et al. (2022). Neurocomputational underpinnings of expected surprise. *Journal of Neuroscience,**42*(3), 474–486. 10.1523/JNEUROSCI.0601-21.2021 34819342 10.1523/JNEUROSCI.0601-21.2021 PMC8802931

[CR36] Faul, F., et al. (2007). G*Power 3: A flexible statistical power analysis program for the social, behavioral, and biomedical sciences. *Behavior Research Methods,**39*(2), 175–191. 17695343 10.3758/bf03193146

[CR37] Gesztesi, G., & Pajkossy, P. (2025). Wink or blush? Pupil-linked phasic arousal signals both change and uncertainty during assessment of changing environmental regularities. *Cognition*, *264*, 106256. 10.1016/j.cognition.2025.106256, https://linkinghub.elsevier.com/retrieve/pii/S0010027725001969 10.1016/j.cognition.2025.106256 40683084

[CR38] Glennon, E., et al. (2019). Locus coeruleus activation accelerates perceptual learning. *Brain Research,**1709*, 39–49. 29859972 10.1016/j.brainres.2018.05.048 PMC6274624

[CR39] Recanzone, G. H. (2009). Interactions of auditory and visual stimuli in space and time. *Hearing Research*, *258*(1), 89–99. 10.1016/j.heares.2009.04.009, https://linkinghub.elsevier.com/retrieve/pii/S0378595509000963 10.1016/j.heares.2009.04.009 PMC2787663 19393306

[CR40] Basgol, H., Dayan, P., & Franz, V. H. (2025). Violation of auditory regularities is reflected in pupil dynamics. *Cortex,**183*, 66–86. 39616966 10.1016/j.cortex.2024.10.023

[CR41] Furth, H. G., & Pufall, P. B. (1966). Visual and auditory sequence learning in hearing-impaired children. *Journal of Speech and Hearing Research,**9*(3), 441–449. 10.1044/jshr.0903.441

[CR42] Liao, H.-I., et al. (2016). Human pupillary dilation response to deviant auditory stimuli: Effects of stimulus properties and voluntary attention. *Frontiers in Neuroscience*, *10*. 10.3389/fnins.2016.00043, http://journal.frontiersin.org/Article/10.3389/fnins.2016.00043/abstract 10.3389/fnins.2016.00043 PMC4756168 26924959

[CR43] Reimer, J., et al. (2016). Pupil fluctuations track rapid changes in adrenergic and cholinergic activity in cortex. *Nature Communications,**7*(1), 13289. 10.1038/ncomms13289 27824036 10.1038/ncomms13289 PMC5105162

[CR44] Kremláček, J., et al. (2016). Visual mismatch negativity (vMMN): A review and meta-analysis of studies in psychiatric and neurological disorders. *Cortex. Special Issue: Repetition suppression-an integrative view*, *80*, 76–112. 10.1016/j.cortex.2016.03.017, https://www.sciencedirect.com/science/article/pii/S0010945216300557 10.1016/j.cortex.2016.03.017 27174389

[CR45] de Gee, J. W., Colizoli, O., Kloosterman, N. A., Knapen, T., Nieuwenhuis, S., & Donner, T. H. (2017). Dynamic modulation of decision biases by brainstem arousal systems. *eLife,**6*, e23232. 10.7554/eLife.23232 10.7554/eLife.23232 PMC5409827 28383284

[CR46] Klingner, J., Tversky, B., & Hanrahan, P. (2011). Effects of visual and verbal presentation on cognitive load in vigilance, memory, and arithmetic tasks. *Psychophysiology,**48*(3), 323–332. 10.1111/j.1469-8986.2010.01069.x 20718934 10.1111/j.1469-8986.2010.01069.x

[CR47] Zhao, J., & Luo, Y. (2017). Statistical regularities guide the spatial scale of attention. *Attention, Perception, & Psychophysics,**79*(1), 24–30. 10.3758/s13414-016-1233-1 10.3758/s13414-016-1233-1 27844342

[CR48] Zhao, J., Al-Aidroos, N., & Turk-Browne, N. B. (2013). Attention is spontaneously biased toward regularities. *Psychological Science,**24*(5), 667–677. 10.1177/0956797612460407 23558552 10.1177/0956797612460407 PMC3734864

[CR49] Krishnamurthy, K., et al. (2017). Arousal-related adjustments of perceptual biases optimize perception in dynamic environments. *Nature Human Behaviour,**1*(6), 0107. 10.1038/s41562-017-0107 29034334 10.1038/s41562-017-0107 PMC5638136

[CR50] Andersen, L. M., & Lundqvist, D. (2019). Somatosensory responses to nothing: An MEG study of expectations during omission of tactile stimulations. *NeuroImage*, *184*, 78–89. 10.1016/j.neuroimage.2018.09.014, https://www.sciencedirect.com/science/article/pii/S1053811918307985 10.1016/j.neuroimage.2018.09.014 30213774

[CR51] Fink, L., et al. (2024). From pre-processing to advanced dynamic modeling of pupil data. *Behavior Research Methods,**56*(3), 1376–1412. 37351785 10.3758/s13428-023-02098-1 PMC10991010

[CR52] Emberson, L. L., Conway, C. M., & Christiansen, M. H. (2011). Timing is everything: Changes in presentation rate have opposite effects on auditory and visual implicit statistical learning. *Quarterly Journal of Experimental Psychology,**64*(5), 1021–1040. 10.1080/17470218.2010.538972 10.1080/17470218.2010.538972 21347988

[CR53] Liao, H.-I., et al. (2016). Correspondences among pupillary dilation response, subjective salience of sounds, and loudness. *Psychonomic Bulletin & Review,**23*, 412–425. 26163191 10.3758/s13423-015-0898-0

[CR54] Wang, L., et al. (2021). Hemodynamic response varies across tactile stimuli with different temporal structures. *Human Brain Mapping,**42*(3), 587–597. 10.1002/hbm.25243 33169898 10.1002/hbm.25243 PMC7814760

[CR55] Guy, M. W., et al. (2021). Peak selection and latency jitter correction in developmental event-related potentials. *Developmental Psychobiology*, *63*(7), e22193. 10.1002/dev.22193, https://www.ncbi.nlm.nih.gov/pmc/articles/PMC8978110/10.1002/dev.22193 PMC8978110 34674252

[CR56] Sabio-Albert, M., Fuentemilla, L., & Pérez-Bellido, A. (2025). Anticipating multisensory environments: Evidence for a supra-modal predictive system. *Cognition,**254*, 105970. 39368349 10.1016/j.cognition.2024.105970

[CR57] Megemont, M., McBurney-Lin, J., & Yang, H. (2022). Pupil diameter is not an accurate real-time readout of locus coeruleus activity. *eLife,**11*, e70510. 10.7554/eLife.70510 35107419 10.7554/eLife.70510 PMC8809893

[CR58] Lisi, M., Bonato, M., & Zorzi, M. (2015). Pupil dilation reveals top-down attentional load during spatial monitoring. *Biological Psychology*, *112*, 39–45. 10.1016/j.biopsycho.2015.10.002, https://www.sciencedirect.com/science/article/pii/S0301051115300636 10.1016/j.biopsycho.2015.10.002 26472280

[CR59] Winn, M. B., Wendt, D., Koelewijn, T., & Kuchinsky, S. E. (2018). Best practices and advice for using pupillometry to measure listening effort: An introduction for those who want to get started. *Trends in Hearing*, *22*. 10.1177/2331216518800869 PMC6166306 30261825

[CR60] Nassar, M. R., et al. (2012). Rational regulation of learning dynamics by pupil-linked arousal systems. *Nature Neuroscience,**15*(7), 1040–1046. 10.1038/nn.3130 22660479 10.1038/nn.3130 PMC3386464

[CR61] Grundei, M., et al. (2023). EEG mismatch responses in a multimodal roving stimulus paradigm provide evidence for probabilistic inference across audition, somatosensation, and vision. *Human Brain Mapping,**44*(9), 3644–3668. 10.1002/hbm.26303 37067073 10.1002/hbm.26303 PMC10203815

[CR62] Van der Stoep, N., et al. (2021). The additive nature of the human multisensory evoked pupil response. *Scientific Reports,**11*(1), 707. 10.1038/s41598-020-80286-1 33436889 10.1038/s41598-020-80286-1 PMC7803952

[CR63] Barascud, N., et al. (2016). Brain responses in humans reveal ideal observer-like sensitivity to complex acoustic patterns. *Proceedings of the National Academy of Sciences*, *113*(5). 10.1073/pnas.1508523113 10.1073/pnas.1508523113 PMC4747708 26787854

[CR64] Grujic, N., Polania, R., & Burdakov, D. (2024). Neurobehavioral meaning of pupil size. *Neuron*, *112*(20), 3381–3395. 10.1016/j.neuron.2024.05.029, www.cell.com/neuron/abstract/S0896-6273(24)00406-9 10.1016/j.neuron.2024.05.029 38925124

[CR65] Éltetö, N., et al. (2022). Tracking human skill learning with a hierarchical Bayesian sequence model. *PLoS Computational Biology,**18*(11), e1009866. 36449550 10.1371/journal.pcbi.1009866 PMC9744313

[CR66] Pajkossy, P., Gesztesi, G., & Racsmány, M. (2023). How uncertain are you? Disentangling expected and unexpected uncertainty in pupil-linked brain arousal during reversal learning. *Cognitive, Affective, & Behavioral Neuroscience,**23*(3), 578–599. 10.3758/s13415-023-01072-w 10.3758/s13415-023-01072-w PMC10390386 36823250

[CR67] Pan, J., Klímová, M., et al. (2022). Arousal-based pupil modulation is dictated by luminance. *Scientific Reports,**12*(1), 1390. 10.1038/s41598-022-05280-1 35082319 10.1038/s41598-022-05280-1 PMC8792027

[CR68] Pan, J., Sun, X., et al. (2024). The effects of emotional arousal on pupil size depend on luminance. *Scientific Reports,**14*(1), 21895. 10.1038/s41598-024-70895-5 39300137 10.1038/s41598-024-70895-5 PMC11412980

[CR69] Binda, P., et al. (2025). Pupillometric signature of implicit learning of statistical regularities. *Current Biology*, *35*(6), 1431-1435e2. 10.1016/j.cub.2025.02.011, https://linkinghub.elsevier.com/retrieve/pii/S0960982225001459 10.1016/j.cub.2025.02.011 PMC11951917 40049171

[CR70] Binda, P., Pereverzeva, M., & Murray, S. O. (2013). Attention to bright surfaces enhances the pupillary light reflex. *Journal of Neuroscience*, *33*(5), 2199–2204. 10.1523/JNEUROSCI.3440-12.2013, https://www.jneurosci.org/content/33/5/2199 10.1523/JNEUROSCI.3440-12.2013 PMC6619119 23365255

[CR71] Dayan, P., & Yu, A. J. (2006). Phasic norepinephrine: A neural interrupt signal for unexpected events. *Network Computation in Neural Systems,**17*(4), 335–350. 10.1080/09548980601004024 17162459 10.1080/09548980601004024

[CR72] Murphy, P. R., et al. (2014). Pupil diameter covaries with BOLD activity in human locus coeruleus. *Human Brain Mapping,**35*(8), 4140–4154. 10.1002/hbm.22466 24510607 10.1002/hbm.22466 PMC6869043

[CR73] Paavilainen, P. (2013). The mismatch-negativity (MMN) component of the auditory event-related potential to violations of abstract regularities: A review. *International Journal of Psychophysiology*, *88*(2), 109–123. 10.1016/j.ijpsycho.2013.03.015, https://linkinghub.elsevier.com/retrieve/pii/S0167876013000652 10.1016/j.ijpsycho.2013.03.015 23542165

[CR74] Zhang, Q., et al. (2022). Visual selective attention P300 source in frontal-parietal lobe: ERP and fMRI study. *Brain Topography,**35*(5), 636–650. 10.1007/s10548-022-00916-x 36178537 10.1007/s10548-022-00916-x

[CR75] Frost, R., et al. (2015). Domain generality versus modality specificity: the paradox of statistical learning. *Trends in Cognitive Sciences*, *19*(3), 117–125. 10.1016/j.tics.2014.12.010, https://linkinghub.elsevier.com/retrieve/pii/S1364661314002770 10.1016/j.tics.2014.12.010 PMC4348214 25631249

[CR76] Vallat, R. (2018). Pingouin: statistics in Python. *Journal of Open Source Software,**3*(31), 1026.

[CR77] Canale, R. (2022). The importance of statistical learning. *Nature Reviews Psychology,**1*(2), 68–68. 10.1038/s44159-021-00010-2 40357017 10.1038/s44159-021-00010-2 PMC12068857

[CR78] Jordan, R. (2024). The locus coeruleus as a global model failure system. *Trends in Neurosciences,**47*(2), 92–105. 38102059 10.1016/j.tins.2023.11.006

[CR79] Bianco, R., Ptasczynski, L. E., & Omigie, D. (2020). Pupil responses to pitch deviants reflect predictability of melodic sequences. *Brain and Cognition*, *138*, 103621. 10.1016/j.bandc.2019.103621, https://linkinghub.elsevier.com/retrieve/pii/S0278262619303185 10.1016/j.bandc.2019.103621 31862512

[CR80] Southwell, R., & Chait, M. (2018). Enhanced deviant responses in patterned relative to random sound sequences. *Cortex*, *109*, 92–103. 10.1016/j.cortex.2018.08.032, https://linkinghub.elsevier.com/retrieve/pii/S0010945218302843 10.1016/j.cortex.2018.08.032 PMC6259587 30312781

[CR81] Southwell, R., et al. (2017). Is predictability salient? A study of attentional capture by auditory patterns. *Philosophical Transactions of the Royal Society B: Biological Sciences,**372*(1714), 20160105. 10.1098/rstb.2016.0105 10.1098/rstb.2016.0105 PMC5206273 28044016

[CR82] Larsen, R. S., & Waters, J. (2018). Neuromodulatory correlates of pupil dilation. *Frontiers in Neural Circuits,**12*, 21. 10.3389/fncir.2018.00021 29593504 10.3389/fncir.2018.00021 PMC5854659

[CR83] Planton, S., & Dehaene, S. (2021). Cerebral representation of sequence patterns across multiple presentation formats. *Cortex*, *145*, 13–36. 10.1016/j.cortex.2021.09.003, https://www.sciencedirect.com/science/article/pii/S0010945221003038 10.1016/j.cortex.2021.09.003 34673292

[CR84] Mathôt, S., et al. (2013). The pupillary light response reveals the focus of covert visual attention. *Plos ONE,**8*(10), e78168. 10.1371/journal.pone.0078168 24205144 10.1371/journal.pone.0078168 PMC3812139

[CR85] Bouret, S., & Sara, S. J. (2005). Network reset: a simplified overarching theory of locus coeruleus noradrenaline function. *Trends in Neurosciences,**28*(11), 574–582. 16165227 10.1016/j.tins.2005.09.002

[CR86] Joshi, S., & Gold, J. I. (2020). Pupil size as a window on neural substrates of cognition. *Trends in Cognitive Sciences*, *24*(6), 466–480. 10.1016/j.tics.2020.03.005, https://linkinghub.elsevier.com/retrieve/pii/S1364661320300802 10.1016/j.tics.2020.03.005 PMC7271902 32331857

[CR87] Joshi, S., et al. (2016). Relationships between pupil diameter and neuronal activity in the locus coeruleus, colliculi, and cingulate cortex. *Neuron*, *89*(1), 221–234. 10.1016/j.neuron.2015.11.028, https://linkinghub.elsevier.com/retrieve/pii/S089662731501034X 10.1016/j.neuron.2015.11.028 PMC4707070 26711118

[CR88] Zhao, S., et al. (2019). Pupil-linked phasic arousal evoked by violation but not emergence of regularity within rapid sound sequences. *Nature Communications,**10*(1), 4030. 10.1038/s41467-019-12048-1 31492881 10.1038/s41467-019-12048-1 PMC6731273

[CR89] Rigato, S., Rieger, G., & Romei, V. (2016). Multisensory signalling enhances pupil dilation. *Scientific Reports,**6*(1), 26188. 10.1038/srep26188, https://www.nature.com/articles/srep26188 10.1038/srep26188 PMC4870616 27189316

[CR90] Durrant, S. J., Cairney, S. A., & Lewis, P. A. (2016). Cross-modal transfer of statistical information benefits from sleep. *Cortex*, *78*, 85–99. 10.1016/j.cortex.2016.02.011, https://linkinghub.elsevier.com/retrieve/pii/S0010945216300247 10.1016/j.cortex.2016.02.011 27017231

[CR91] Simpson, H. M. (1969). Effects of a task-relevant response on pupil size. *Psychophysiology*, *6*(2). 10.1111/j.1469-8986.1969.tb02890.x 5345494

[CR92] D’Ascenzo, S., et al. (2018). Visual versus auditory Simon effect: A behavioural and physiological investigation. *Quarterly Journal of Experimental Psychology,**71*(4), 917–930. 10.1080/17470218.2017.1307429 10.1080/17470218.2017.1307429 28293982

[CR93] Sylvain Sirois and Julie Brisson. (2014). Pupillometry. *Wiley Interdisciplinary Reviews: Cognitive Science,**5*(6), 679–692. 26308873 10.1002/wcs.1323

[CR94] Liu, W., et al. (2024). Linear integration of multisensory signals in the pupil. *Psychophysiology,**61*(2), e14453. 10.1111/psyp.14453 37813676 10.1111/psyp.14453

[CR95] Teh, Y. W. (2006). A hierarchical Bayesian language model based on Pitman-Yor processes. In *Proceedings of the 21st International Conference on Computational Linguistics and 44th Annual Meeting of the Association for Computational Linguistics*, (pp. 985–992).

[CR96] Cai, Y., et al. (2024). Open-DPSM: An open-source toolkit for modeling pupil size changes to dynamic visual inputs. *Behavior Research Methods,**56*(6), 5605–5621. 10.3758/s13428-023-02292-1 38082113 10.3758/s13428-023-02292-1 PMC11335788

[CR97] Mridha, Z., et al. (2021). Graded recruitment of pupil-linked neuromodulation by parametric stimulation of the vagus nerve. *Nature Communications,**12*(1), 1539. 10.1038/s41467-021-21730-2 33750784 10.1038/s41467-021-21730-2 PMC7943774

