# Supplementary material for: Modality-general sensitivity of pupil responses to regularity violations
Source: Cogn Affect Behav Neurosci. 2026 Apr 7;26(4):1684–705. doi: 10.3758/s13415-026-01423-3 (PMC13385247; doi:10.3758/s13415-026-01423-3)
Supplement: Supplementary file 1 — (pdf 4083 KB) [file 13415_2026_1423_MOESM1_ESM.pdf]

Modality-General Sensitivity of Pupil Responses to Regularity Violations

Supplementary Materials

Hamit Basgol[1, 2, 3], Florian Raab[3], Peter Dayan[1, 4], and Volker H. Franz[1, 2]

[1]Department of Computer Science, University of Tübingen, Tübingen, Germany

[2]Experimental Cognitive Science, University of Tübingen, Tübingen, Germany

[3]The Graduate Training Centre of Neuroscience, University of Tübingen, Tübingen, Germany

[4]Max Planck Institute for Biological Cybernetics, Tübingen, Germany

S1 Analysis of Sustained Pupil Responses

S1.1 Experiments 1 and 2:

To explore potential differences between REG5 and RAND5, we analysed pupil size data 6 s after the start of each trial. Baseline pupil size was calculated by averaging pupil measurements taken during the first 0.25 s (due to the missing pupil size measurements in the inter-trial interval) after trial onset (i.e., during the first repetition of a possible regularity). We then subtracted this baseline value from pupil sizes recorded during the 6 s epoch to assess the influence of the pattern types on pupil size. We compared pupil sizes in REG5 and RAND5. As shown in Figure S1a, there is no considerable difference in pupil size between the two conditions. However, there appears to be a gradual decline in pupil size during the REG5 trials. Bayesian analysis supports this evidence, such that the BF_{10} exceeds 3 at around 5.48 s, suggesting moderate evidence in favour of a difference. However, this evidence decreases, with the BF_{10} dropping below 3 at 5.68 s (see Figure S1a).

In Experiment 2, we observed that pupil responses to regular patterns spontaneously increased, possibly as a proxy for the effort participants exerted on the task (see Figure S1b). This observation suggests that the detection task in Experiment 2 was possibly more effortful than the gap detection task in Experiment 1. This aspect of the results was not investigated, as it was beyond the scope of the paper.

S1.2 Experiment 3:

In Experiment 3, we presented participants with visual and auditory regularities. Pupil responses to consistent auditory patterns were greater than those to visual patterns (see Figure S2). In light of accounts that sustained pupil size is a proxy of effort (Van der Wel and Van Steenbergen, 2018), this result may indicate that processing of frequencies (in the scope of the gap detection task) might require more effort.

Baseline pupil size was calculated by averaging pupil measurements taken during the 1-second interval before trial onset. This baseline value was then subtracted from the pupil sizes recorded during the trial to analyse the effect of presentation modality on pupil size.

Figure S1

Pupil responses to pattern types.

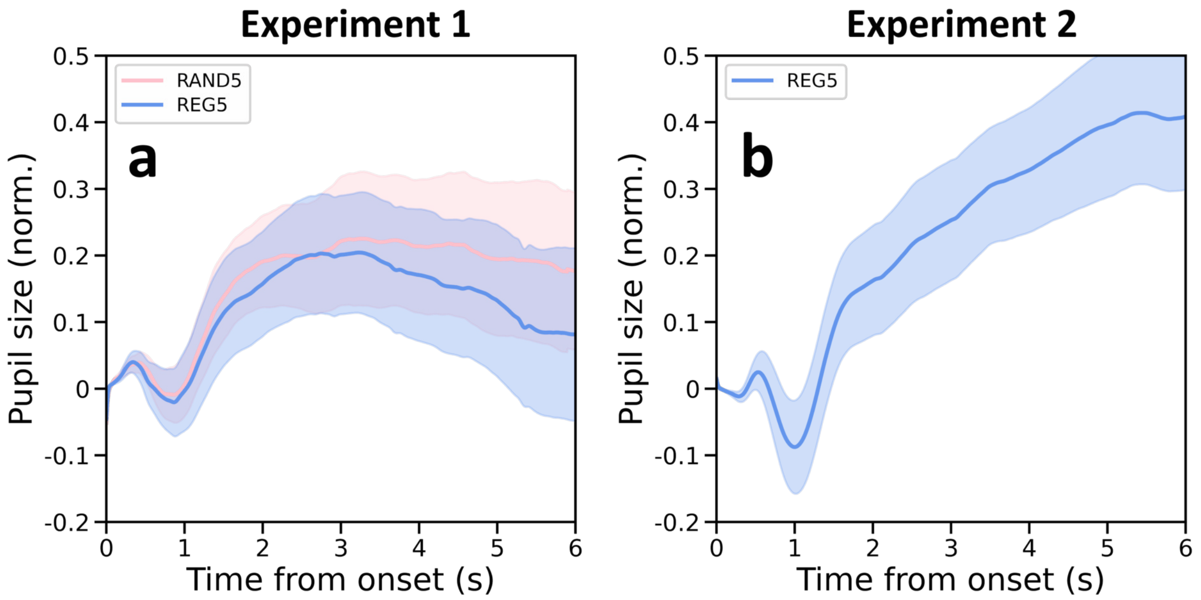

Note. Sustained pupil responses in Experiments 1 and 2. **(a)** Experiment 1. Pupil dilation responses to pattern types. Average normalised pupil size over time in no-transition control conditions. These conditions led to similar changes in pupil size. However, there appears to be a gradual decline in pupil size for REG5. **(b)** Experiment 2. Shaded areas indicate the between-participant standard error of the means.

Figure S2

Pupil responses to REG5 across modalities.

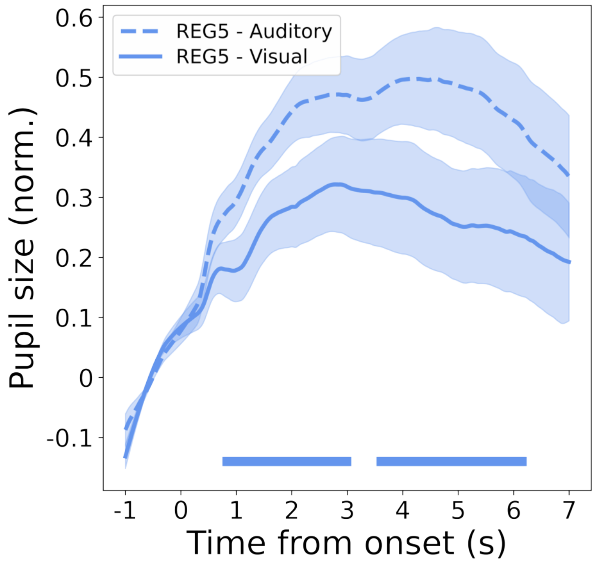

Note. Sustained pupil responses in Experiment 3. Pupil dilation responses to REG5 across modalities. Average normalised pupil size over time in no-transition control conditions. These conditions led to different pupil size changes, such that the auditory regular patterns elicited a heightened pupil response compared to the visual regular patterns. Coloured horizontal lines indicate regions where cluster-level statistics $p < 0.05$ for the difference between visual and auditory modalities. Shaded areas indicate the between-participant standard error of the means.

S2 Comparisons of Experiments

We show all experiments and the same conditions on the same figure (see Figure S3).

Figure S3

Pupil baseline and dilations across experiments.

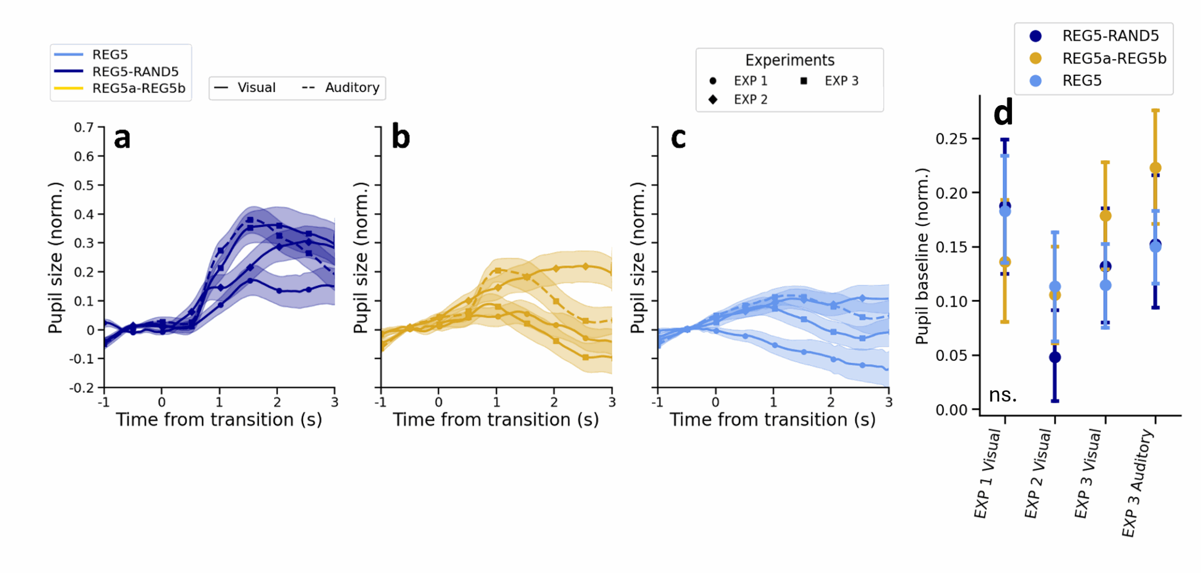

Note. Pupil baseline and dilations across experiments. **(a)** pupil responses in REG5-RAND5, **(b)** in REG5*a*-REG5b and **(c)** in REG5, **(d)** pupil baselines that are calculated by averaging 1 sec before the transition. Shaded areas and error bars indicate the between-participant standard error of the means.

S3 Statistics of Sequences

To quantify the degree of surprise elicited by transitions in the stimulus sequences, we analysed participants' trial-wise surprise using a hierarchical Chinese Restaurant Process (HCRP) sequence model (Figure S4; Éltető et al., 2022; Teh, 2006). This model provides a domain-general account of sequence learning by capturing statistical regularities across multiple contextual depths without relying on modality-specific assumptions. For each experiment, we analysed the surprisal associated with transitions by fitting the model to the sequence of trials experienced by each participant, yielding trial-wise surprise estimates.

Figure S4

Statistics of sequences.

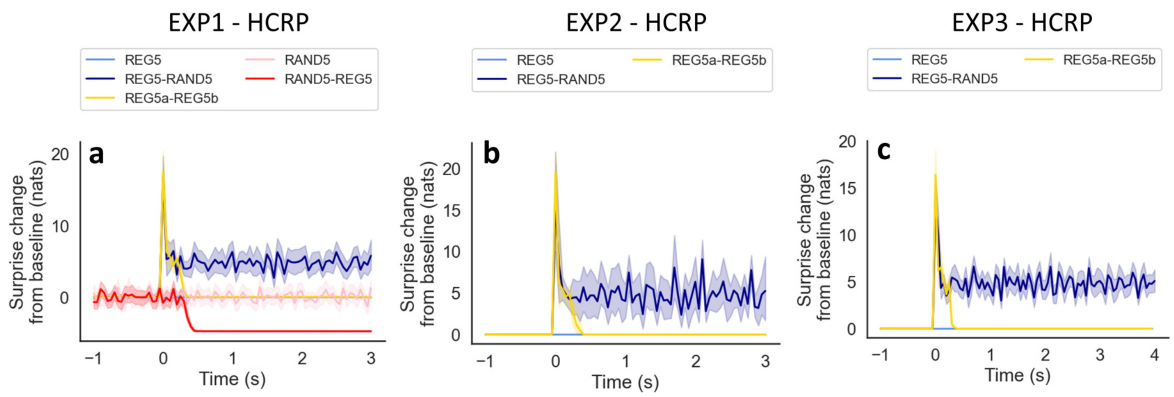

Note. Surprise values were estimated using a hierarchical Chinese Restaurant Process (HCRP) sequence model (Éltetö et al., 2022), which was fitted to the sequence of trials experienced by participants during an experimental session. To correct for baseline differences, the average surprise computed over the 1-second interval preceding the transition (from −1 to 0 s) was calculated and subtracted from the surprise values of each trial. **(a)** shows analyses for Experiment 1, **(b)** for Experiment 2 and **(c)** for Experiment 3. Shaded areas indicate the between-participant standard error of the mean.

S4 Comparison with the Previous Study

In our previous work, we investigated pupil dilation responses to complex auditory sequences (complexity of 10); violations of regularities, both by random patterns (REG10-RAND10) and by novel regularities (REG10a-REG10b), led to pupil dilation responses, as shown in Figure S5a. The emergence of regularities from a random sequence (RAND10-REG10) did not yield an increase relative to the random baseline (RAND10).

We obtained a similar pattern of results when using shorter sequences (length 5), as shown in Figure S5b. While most conditions mirrored the findings from the more complex sequences, the pupil dilation responses observed for the violation of a regularity by a novel regularity (REG5a-REG5b) were notably smaller in magnitude. These observations on pupil response to regularity changes were replicated even when the nature of the task was modified to a more engaging shape detection task (see Figure S5c).

We performed a direct comparison of pupil dilation responses to structured stimuli across auditory (frequencies) and visual (changing dot positions) modalities, as in Figures S5d and S5e. When a regular pattern was violated by a random one (REG5-RAND5), the pupil responses were similar across modalities and consistent with our previous findings (compare REG5-RAND5 in Figures S5d and S5e with Figure S5a). In contrast, when a presented

regularity was violated by a novel one (REG5a-REG5b), the pupil responses diverged between the auditory and visual modalities (compare REG5a-REG5b in Figures S5d and S5e with Figure S5a).

Figure S5

The comparison with the previous study.

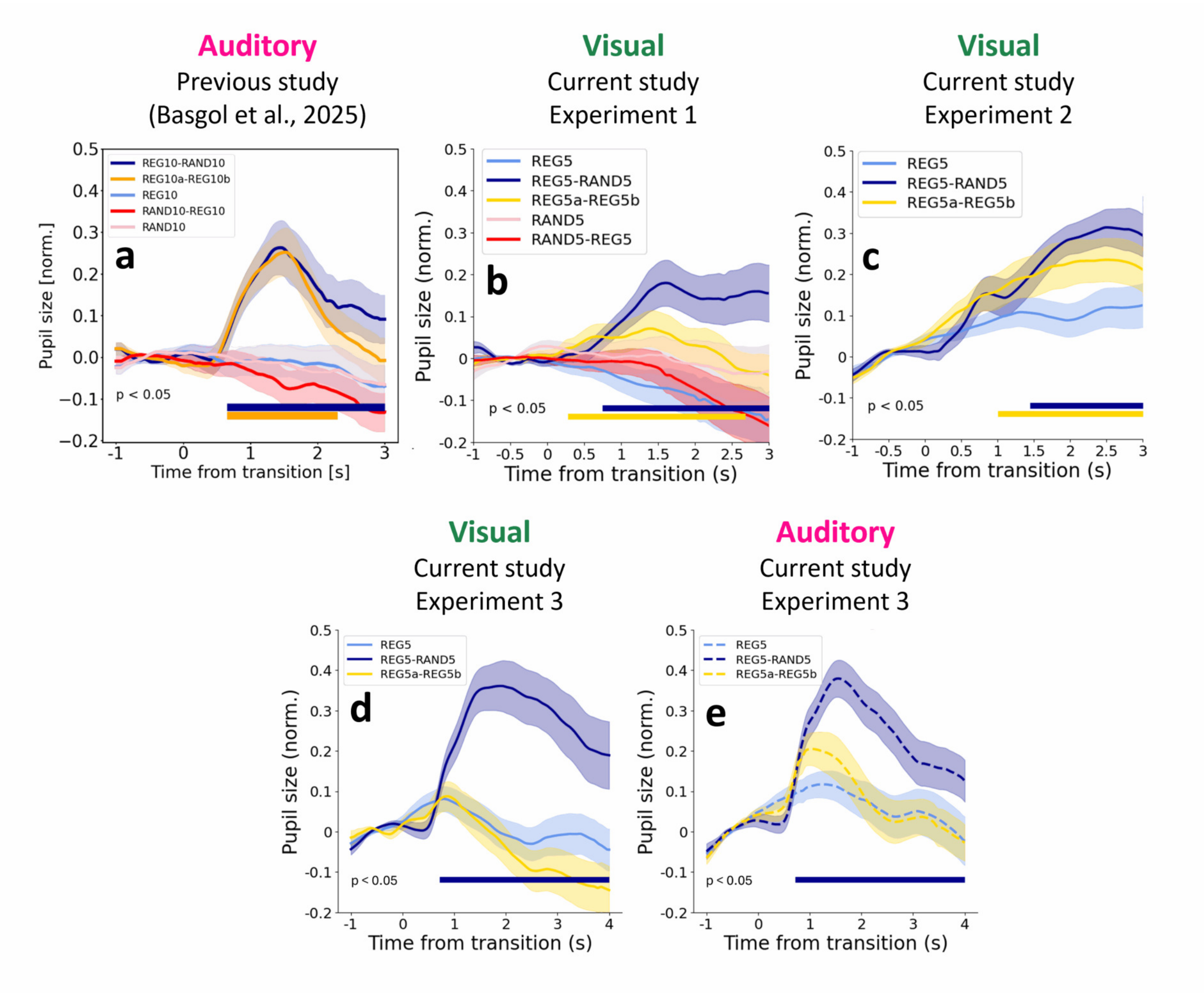

Note. The comparison of the current results of pupil dilation responses with the previous study. **(a)** In our previous study, Basgol et al., 2025, we investigated pupil dilation responses to regularity violations, but in sequences of length 10, with items being short presentations of particular pure-tone frequencies. Violations of regularities with random patterns (REG10-RAND10) and novel regularities (REG10a-REG10b) led to an increase. The emergence of regularities from random patterns (RAND10-REG10) did not result in a significant increase compared to the baseline (RAND10). **(b)** Similar results were obtained here with shorter sequences of length 5. However, smaller pupil dilation responses were observed for the violation of regularities by novel regularities (REG5a-REG5b). **(c)** Similar observations were obtained when the task was changed to a more engaging shape detection task. **(d, e)** We directly compared pupil dilation responses when structured stimuli were presented in auditory, as frequencies, and vision, as changing dot positions. Similar pupil responses were obtained when random patterns violated presented regularities (compare REG5-RAND5 in **d, e** with **a**). In contrast, pupil responses diverged when novel regularities violated presented regularities (compare REG5a-REG5b in **d, e** with **a**). Baseline pupil responses (i.e., REG5) tend to fluctuate. Shaded areas indicate the between-participant standard error of the means. Coloured horizontal lines indicate regions where cluster-level statistics $p < .05$. (a) Reproduced from the supplementary material of Basgol, H., Dayan, P., & Franz, V. H. (2025). Violation of auditory regularities is reflected in pupil dynamics. Cortex, 183, 66-86. CC BY 4.0.

We conducted exploratory analyses to identify the factors that may contribute to the missing effect in REG5a-REG5b (see Figures S5d and S5e). One factor we considered was pupil baseline, as it is known that baseline measurements can obscure increases in pupil size (Relaño-Iborra et al., 2022). Our analysis, however, revealed that the pupil baseline in Experiment 3 was similar to the pupil baseline observed in Experiment 1 (see Figure S3). Furthermore, pupil size increased in the REG5-RAND5 condition, which rules out the potential influence of pupil baseline.

Another factor is experiment duration, which has been associated with a reduction in pupil size increase (McLaughlin et al., 2023). We also observed that the duration of the experiment influenced pupil size, with regularity violations in initial blocks causing a slightly larger increase. However, even the earliest trials did not demonstrate effects consistent with previous experiments (see REG5a-REG5b in Figure S3).

Participants may also have implicitly learned when the transition occurred (Akdoğan et al., 2016), allowing them to modulate their effort in response to changing task demands. This modulation could have produced a gradual increase in pupil size around the time of transitions, followed by a reduction in REG5 (see Figure S8). It may also explain the reduction in pupil size observed in REG5a–REG5b, resulting from more efficient effort regulation.

It is important to note that the absence of time-series pupil size increase does not imply that REG5a-REG5b did not affect pupil size; in fact, pupil dilation events (presented in the main text) have increased due to these transitions.

S5 Pupil Event Rate Analysis

A typical pupillometry analysis involves averaging event-related pupil responses across trials. Still, this method can misrepresent peak amplitudes and response latencies (Fink et al., 2024), as is also the case with signals such as functional magnetic resonance imaging (fMRI) and EEG (Guy et al., 2021; Wang et al., 2021).

To address these problems, we extracted pupil events (i.e., dilations and constrictions) from continuous pupil data. These were identified by analysing changes in pupil size slopes. This method enabled a more detailed analysis of condition and modality differences.

In Experiments 1 and 2, we observed differences between the transition conditions and the no-transition control condition. However, these differences did not survive the cluster-based permutation test (except for the strong constriction response in the

REG5-RAND5 condition of Experiment 1). On the other hand, increasing statistical power by combining Experiments 1 and 2 (though note that differences exist across experiments that could increase variability) revealed a strong response in the REG5-RAND5 condition (in terms of event rates and magnitudes) and a potential difference in the REG5a-REG5b condition (in terms of event rates), which was later confirmed in Experiment 3 (see the main text for the discussion).

Figure S6

Pupil event rate analyses for Experiments 1 and 2.

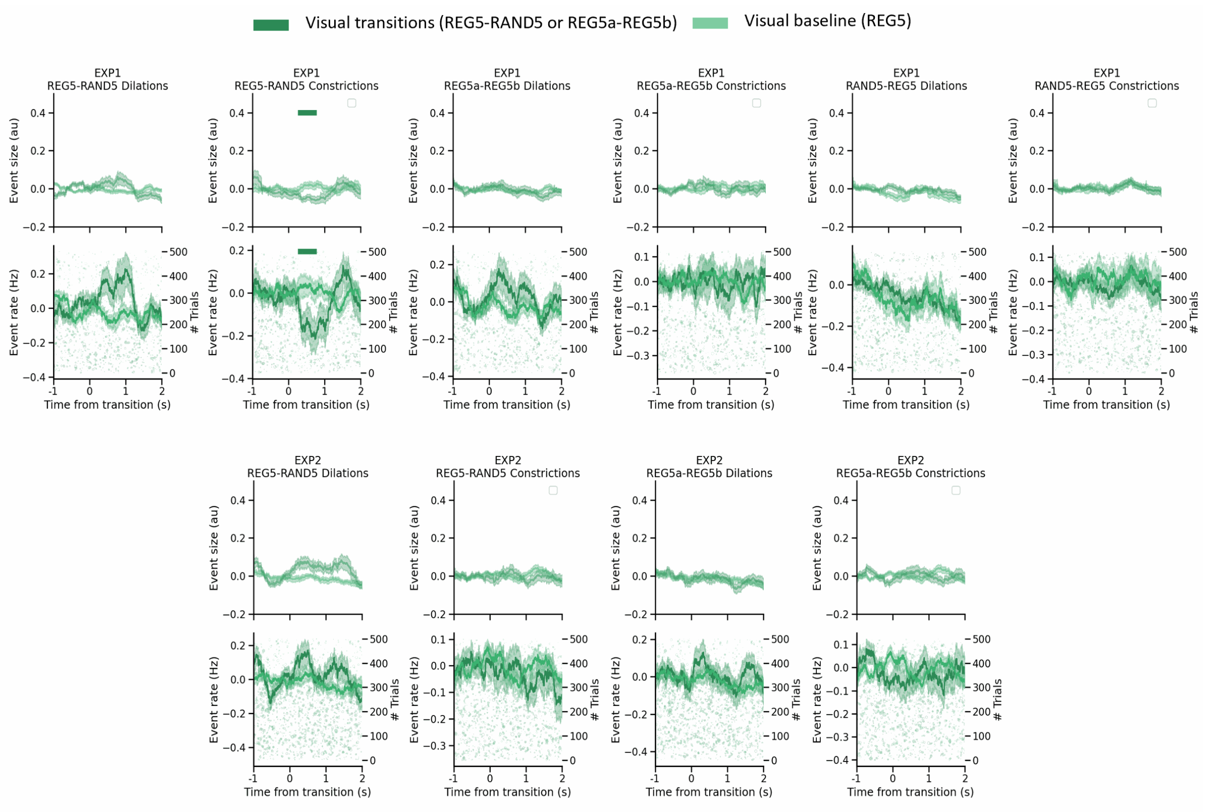

Note. Pupil event rate analyses for Experiments 1 and 2. Pupil dilation and constriction rates were computed using a running average with a 500-ms window. Overall, dilation rates for REG5-RAND5 and REG5a-REG5b slightly increased (this increase became significant after combining Experiments 1 and 2, as described in the main text). On the other hand, the transition from RAND5 to REG5 did not result in a considerable change. Coloured horizontal lines indicate regions where cluster-level statistics $p < 0.05$ between each transition condition (shown as dark green) and the no-transition control (light green). Shaded areas represent between-participant standard error of the means.

S6 Control Analyses for Correlations

We conducted correlations across modalities based on scalar measures of pupil size, as presented in the main text. We also conducted a control analysis to estimate whether these correlations are based on a common variable that could be associated with the temporal dynamics of the experiment (remember that trial pairs occur in the same order within the

block). We conducted control analyses to address the potential impact of isomorphic pairs on our results. To do this, we re-ran the analyses with "fake" isomorphic pairs, which were determined as the closest trials in time to the original pair, within the same condition and block.

Out of 32 tests estimating correlations, only one test yielded a BF_{10} greater than 3, which is the correlation of modalities for REG5 (Mr = 0.09, BF_{10} = 12.59, p_{corr} = 0.063, see Tables S1 and S2). No other comparison yielded a $BF_{10} > 3$ (with all other corrected p-values $> .476$).

Table S1

Control Analysis: Correlations of Participants

dv	Statistics Conditions	t	M_r	%95 CI	BF_{10}	p_{corr}	d
Peak size	REG5-RAND5	0.03	0.0	[-0.07, 1]	0.46	1.0	0.01
	REG5a-REG5b	-0.04	-0.0	[-0.1, 1]	0.46	1.0	0.01
	Transitions	0.67	0.02	[-0.03, 1]	0.57	1.0	0.15
	**REG5**	**2.97**	**0.09**	**[0.04, 1]**	**12.59**	**0.063**	**0.66**
Peak time	REG5-RAND5	-0.21	-0.01	[-0.11, 1]	0.47	1.0	0.05
	REG5a-REG5b	0.25	0.01	[-0.07, 1]	0.48	1.0	0.06
	Transitions	0.28	0.01	[-0.05, 1]	0.48	1.0	0.06
	REG5	0.49	0.02	[-0.05, 1]	0.52	1.0	0.11
Mean size	REG5-RAND5	1.05	0.04	[-0.03, 1]	0.76	1.0	0.24
	REG5a-REG5b	-0.23	-0.01	[-0.1, 1]	0.48	1.0	0.05
	Transitions	1.01	0.04	[-0.02, 1]	0.73	1.0	0.23
	REG5	1.96	0.07	[0.01, 1]	2.26	0.49	0.44
Minimum size	REG5-RAND5	0.51	0.03	[-0.06, 1]	0.52	1.0	0.12
	REG5a-REG5b	0.42	0.02	[-0.06, 1]	0.5	1.0	0.09
	Transitions	0.67	0.02	[-0.04, 1]	0.57	1.0	0.15
	REG5	1.39	0.06	[-0.01, 1]	1.08	1.0	0.31

Note. Individual correlations based on isomorphic pairs of visual and auditory sequences were calculated for each participant (but now after selecting fake pairs for control analyses). The correlations were then compared to 0 using a one-tailed t-test. M_r corresponds to the mean of individual correlations; 95% CI denotes the one=sided confidence interval. The statistical metrics BF_{10}, p_{corr}, and d_z refer, respectively, to the Bayes factor, the corrected p-values of these correlations, and effect sizes.

Table S2

Control Analysis: Correlations across Isomorphic Transitions

dv	Statistics<br>Conditions	r	%95 CI	BF_{10}	p_{corr}
Peak size	REG5-RAND5	-0.14	[-0.51, 0.28]	0.31	1.0
	REG5a-REG5b	-0.44	[-0.72, -0.04]	2.24	0.476
	Transitions	-0.02	[-0.3, 0.27]	0.18	1.0
	REG5	0.32	[0.04, 0.55]	1.96	0.424
Peak time	REG5-RAND5	-0.09	[-0.47, 0.33]	0.27	1.0
	REG5a-REG5b	-0.15	[-0.52, 0.27]	0.32	1.0
	Transitions	-0.04	[-0.32, 0.25]	0.19	1.0
	REG5	0.11	[-0.18, 0.39]	0.24	1.0
Mean size	REG5-RAND5	0.06	[-0.35, 0.45]	0.26	1.0
	REG5a-REG5b	-0.29	[-0.62, 0.12]	0.63	1.0
	Transitions	0.13	[-0.16, 0.4]	0.26	1.0
	REG5	0.28	[-0.0, 0.52]	1.1	0.748
Minimum size	REG5-RAND5	0.11	[-0.31, 0.49]	0.29	1.0
	REG5a-REG5b	-0.11	[-0.49, 0.31]	0.29	1.0
	Transitions	0.12	[-0.17, 0.39]	0.25	1.0
	REG5	0.21	[-0.08, 0.47]	0.49	1.0

Note. Correlations were calculated based on the mean of visual and auditory transitions (but now after selecting fake pairs for control analyses), reflecting group-level responses. r denotes the correlation coefficient; 95% CI denotes the confidence interval. The statistical metrics BF_{10} and p_{corr} refer, respectively, to the Bayes factor and the corrected p-values of these correlations.

S7 Correlations of Modalities based on Pupil Events

We calculated scalar measures of pupil size for isomorphic trial pairs. Similar to this analysis, we calculated the number of dilation events and the sum of event size, and computed correlations between these values across isomorphic trial pairs. Interestingly, the number of dilation events was not correlated; however, the total size of events was correlated in both transition and baseline conditions across modalities (see Tables S3 and S4). Although modalities resulted in similar patterns of results (see the main manuscript), they were not correlated across modalities due to the limited range (the maximum number of events in a trial was only 4, and the minimum was 0).

Table S3

Correlations of Participants for Dilation Events

dv	Statistics<br>Conditions	t	M_{r}	%95 CI	BF_{10}	p_{corr}	d
Number of dilation events	REG5-RAND5	2.12	0.1	[0.02, 1]	2.9	0.118	0.47
	REG5a-REG5b	-0.15	-0.01	[-0.09, 1]	0.47	1.0	0.03
	Transitions	0.73	0.02	[-0.02, 1]	0.59	0.76	0.16
	REG5	-0.13	-0.0	[-0.06, 1]	0.47	1.0	0.03
Sum of event size	**REG5-RAND5**	**3.77**	**0.17**	**[0.1, 1]**	**58.71**	**0.005**	**0.84**
	REG5a-REG5b	0.9	0.06	[-0.05, 1]	0.66	0.76	0.2
	**Transitions**	**4.23**	**0.13**	**[0.08, 1]**	**145.28**	**0.002**	**0.95**
	**REG5**	**3.46**	**0.13**	**[0.07, 1]**	**32.06**	**0.008**	**0.77**

Note. Individual correlations based on isomorphic pairs of visual and auditory sequences were calculated for each participant. The correlations were then compared to 0 using a one-tailed t-test. M_r corresponds to the mean of individual correlations; 95% CI denotes the confidence interval from the t-test. Because of the one-tailed t-test, only one tail of the CI is given. The statistical metrics BF_{10}, p_{corr}, and d_z refer, respectively, to the Bayes factor, the corrected p-values of these correlations, and effect sizes.

Table S4

Correlations across Isomorphic Transitions for Dilation Events

dv	Statistics<br>Conditions	r	%95 CI	BF_{10}	p_{corr}
Number of dilation events	REG5-RAND5	0.16	[-0.26, 0.53]	0.33	1.0
	REG5a-REG5b	-0.14	[-0.51, 0.28]	0.31	1.0
	Transitions	0.05	[-0.24, 0.33]	0.19	1.0
	REG5	-0.24	[-0.49, 0.05]	0.65	0.515
Sum of event size	**REG5-RAND5**	**0.51**	**[0.14, 0.76]**	**5.65**	**0.062**
	REG5a-REG5b	0.22	[-0.2, 0.57]	0.42	1.0
	**Transitions**	**0.52**	**[0.28, 0.7]**	**203.08**	**0.001**
	**REG5**	**0.66**	**[0.46, 0.79]**	**> 1000**	**$< .001$**

Note. Correlations were calculated based on the mean of visual and auditory transitions, reflecting group-level responses. r denotes the correlation coefficient; 95% CI denotes the confidence interval. The statistical metrics BF_{10} and p_{corr} refer, respectively, to the Bayes factor and the corrected p-values of these correlations.

S8 Temporal Analyses

We investigated whether the timing within the experiment influences pupil responses to regularity violations and whether these effects are consistent across modalities. To this end, we divided the experiment into three segments: early (blocks 1–2), mid (3–4), and late (5–6), and averaged the time-series pupil size along with associated scalar measures (i.e., peak and mean pupil sizes).

Figure S7 illustrates the temporal dynamics of pupil responses to two types of statistical transitions, REG5–RAND5 and REG5a–REG5b, across experimental blocks. Figures S7 a to c and Figures S7 f to h display time-locked, normalised pupil size traces for early (blocks 1–2), mid (3–4), and late (4–6) segments, separately for each condition. Solid and dashed lines denote the visual and auditory modalities, respectively. In the REG5–RAND5 condition (Figure S7a to c), pupil dilation responses were large and sustained across the session, although a gradual reduction in amplitude was observed over time. By contrast, REG5a-REG5b transitions (Figures S7f to h) elicited smaller responses overall, with a more pronounced decline across blocks, particularly in the visual modality.

Summary metrics of these responses are shown in Figures S7d and e (REG5–RAND5) and Figures S7i and j (REG5a–REG5b), plotting peak and mean pupil size as a function of block group. For REG5–RAND5, both modalities exhibited a similar decline in pupil response over time (Figures S7d and e), consistent with habituation to continuous violations during the experiment. In contrast, for REG5a–REG5b (Figures S7i and j), the auditory modality remained stable across blocks, while the visual modality showed a significant reduction in both peak and mean pupil size. This divergence, indicated by asterisks, reflects a modality-specific sensitivity to transient statistical changes. Note that p-values are not corrected for multiple comparisons. The correction operation (with 12 tests) yielded no test results suggesting a significant difference.

Figure S7

Temporal Analyses of Pupil Responses for Experiment 3.

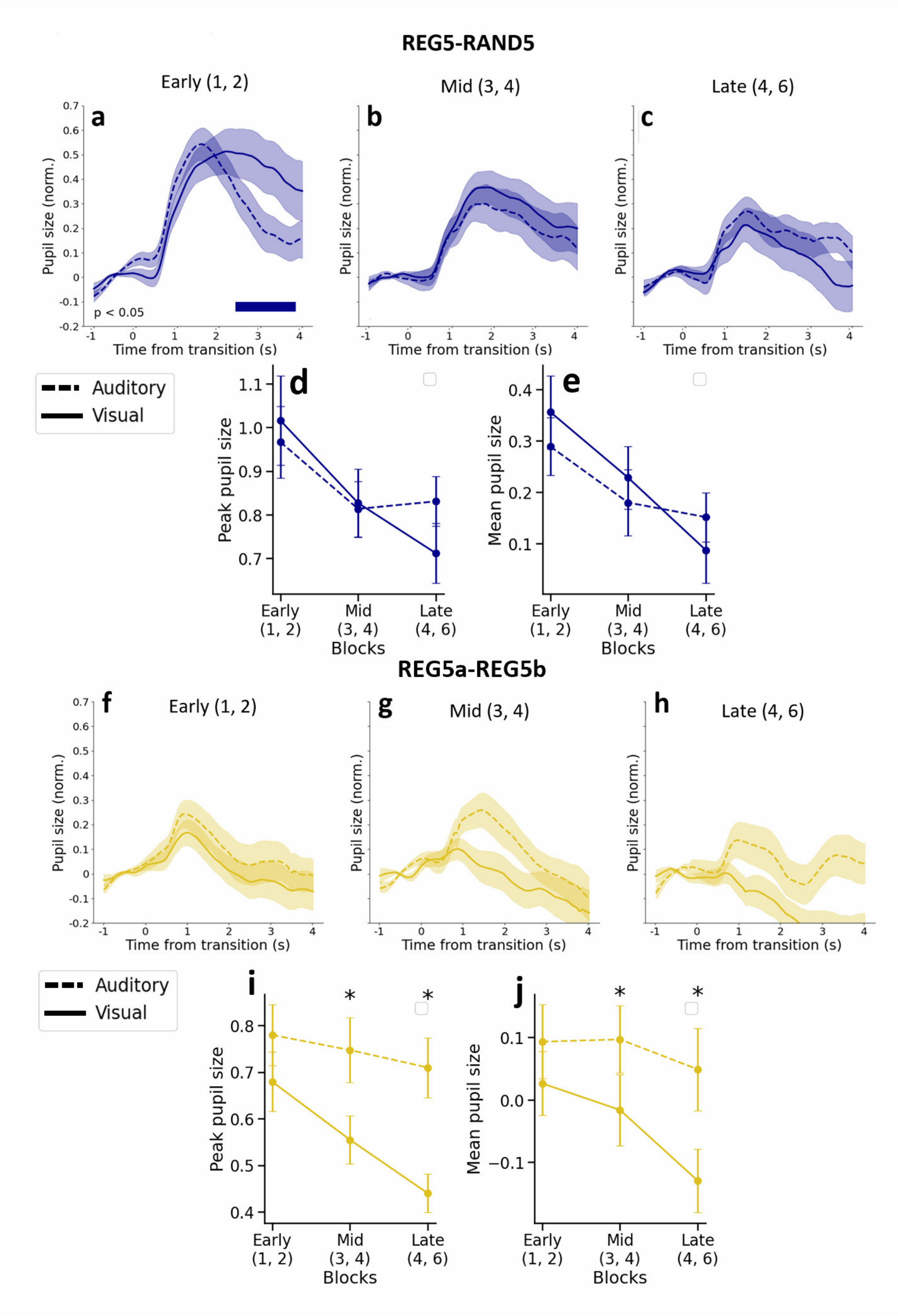

Note. Pupil responses in Experiment 3 across groups of blocks. Panels a-c and f-h show normalised pupil size in the REG5–RAND5 (blue, top row) and REG5a–REG5b (yellow, bottom row) conditions, split by block groups: **(1, 2)** early, **(3, 4)** mid, and **(5, 6)** late. In the REG5–RAND5 condition, pupil dilation remained, though with a gradual reduction in amplitude **(a, c)**. In contrast, REG5a–REG5b transitions evoked smaller pupil dilations overall, especially in the visual modality **(f, h)**. **(d-e)** and **(i, j)** summarise peak and mean pupil sizes across block groups for each condition and modality. In REG5–RAND5, pupil size decreased over time for both modalities without a strong divergence **(d, e)**. In REG5a–REG5b, auditory responses remained stable, whereas visual responses declined more over time **(i, j)**, indicating a modality-specific adaptation to transient statistical violations. Asterisks denote $p < 0.05$ between modalities. Note that p-values are not corrected. Error bars and shaded areas indicate between-participant standard error of the means.

Figure S8

Temporal Analyses of Pupil Responses.

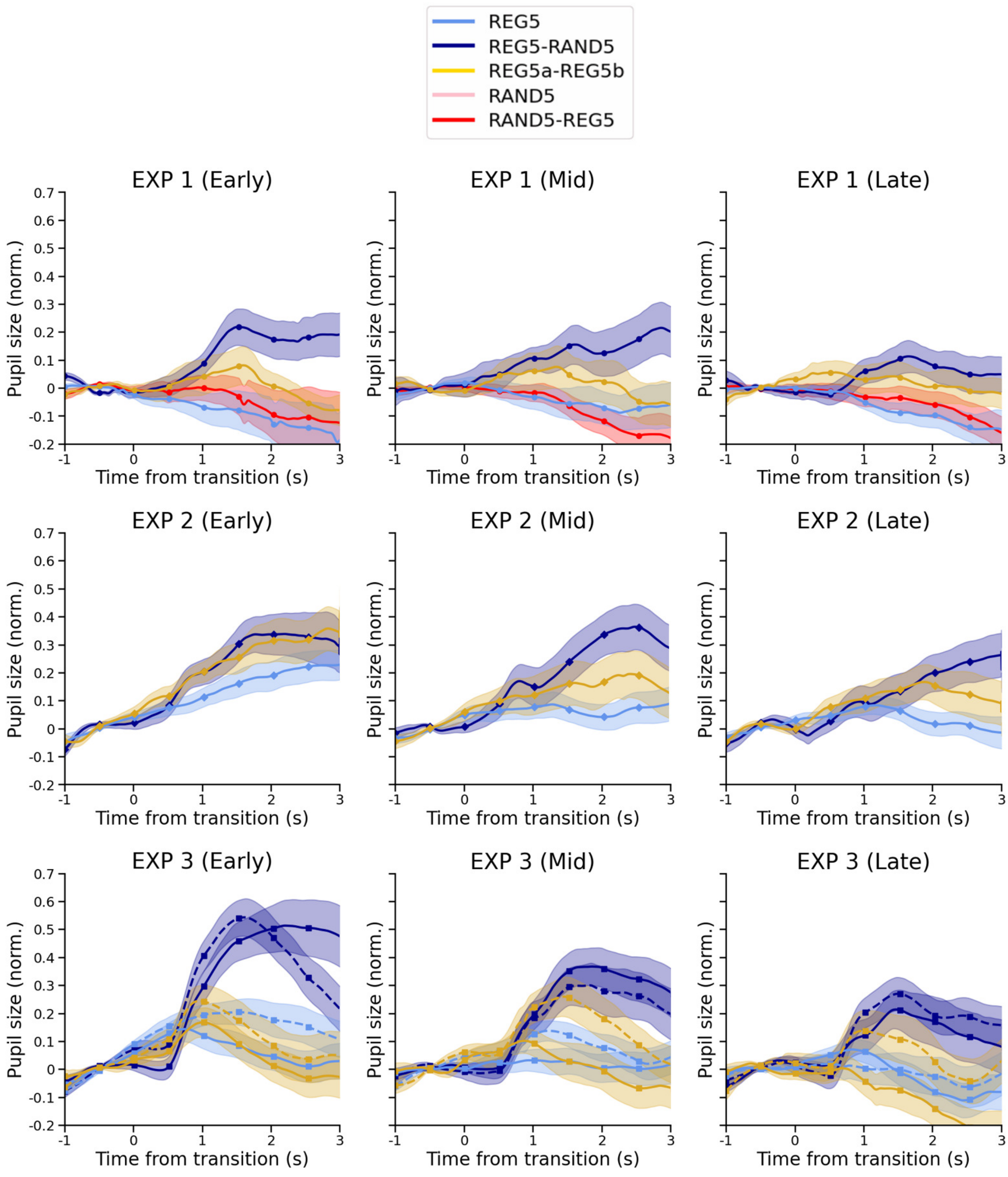

Note. Pupil responses across groups of blocks for all experiments. Pupil responses during different experiments as a function of the time from the transition. The first column shows pupil responses in the early phase of the experiment (1, 2 blocks); the second column shows the mid phase of the experiment (3, 4 blocks), and the last column shows the late phase of the experiment (5, 6 blocks). Error bars and shaded areas indicate between-participant standard error of the means. Error bars and shaded areas indicate between-participant standard error of the means.

S9 Saliency Analyses

Following Conway and Christiansen (2005), we asked participants to judge similarities between items (i.e., tones and dots appearing on a grid) on a continuous scale from 0 to 100 (i.e., 81 randomly selected, pairwise comparisons of 9 items within a given modality). Each item was displayed for 0.5 s, with an inter-presentation interval of 1 s. In a subsequent experiment, participants were presented with short versions of transitions (1 s before and 1 s after the transition, totalling 2 s). They rated the saliency of these transitions using a continuous scale from 0 to 100 (i.e., 48 judgements for visual items and 48 for auditory items, totalling 96).

Participants then participated in the item similarity judgment experiment, where they were asked to assess the similarity between pairs of items. Before the experiment, they were shown the most distant (i.e., dissimilar) items from the dataset (auditory: 222 Hz and 1536 Hz tones; visual: dots in the bottom-left and upper-right corners of the reference grid), and they were asked to scale their similarity judgements accordingly.

All permutations were presented to participants in a random order. We conducted this experiment to design possible exploratory analyses that could estimate representations of items that participants form; however, given confirmed predictions and the strong correlations across modalities, we did not analyse the results of this measurement.

Participants then rated transitions based on their saliency. They were presented with three example trials (one from each condition; REG5, REG5a-REG5b, and REG5-RAND5) and instructed to evaluate the degree of subjective detectability (i.e., saliency) of transitions. Participants were encouraged to respond consistently, reflecting a consistent and ordered relationship. This procedure provided a shared basis for comparing pupil responses observed across modalities (Liao et al., 2016). We first rescaled participants' saliency judgements (in isolation for each modality) to a 0-100 range to minimise the influence of individual scaling biases on overall trends (note that this rescaling was not specified in the preregistration). We then examined correlations of these judgements between modalities at individual and group levels.

We investigated the relationship between participants' pupil responses following the transition and their saliency judgements by running a linear mixed effects model for each modality and time point (using the statsmodels package in Python; Seabold and Perktold,

2010) to predict pupil size (without baseline correction). We based our analysis on all trials without splitting the data into transitions, as participants judged transitions in the same session and were instructed to rate salience in an ordered relationship, independent of conditions. For the sake of completeness, we re-ran the analysis for all conditions and modalities.

We used a linear mixed model to consider the effect of participant-level variables associated with the overall experiment, such as reaction times (RTs), sensitivity (d') and mental effort. We included the following predictors in the models: baseline pupil responses for each trial (averaged over one second before the transition), d', RTs and mental effort reported by participants for gap detection tasks. All variables in the model were normalised to ensure the comparability of coefficients.

We examined participants' saliency judgements for visual and auditory transitions to find out a common perceptual metric for transitions (Joshi et al., 2016). These judgements were correlated (see Figure S9b; REG5-RAND5: $z = 0.13$, $BF_{10} = 5.75$, $p = .009$, 95% CI = [0.04, 0.22], $d_z = 0.65$; REG5a-REG5b: $z = 0.08$, $BF_{10} = 2.45$, $p = .025$, 95% CI = [0.01, 0.16], $d_z = 0.55$). However, the average correlations among participants were only around 0.1, which may be attributed to the experimental structure. In the experiment, participants judged and compared all transitions together, independent of conditions. Indeed, the correlation between saliency judgements for sequences increased when transition trials were considered together ($z = 0.30$, $BF_{10} > 1000$, $p < .001$, 95% CI = [0.23, 0.37], $d_z = 1.96$).

We calculated the average saliency per transition and calculated correlations of these averages. These values were highly correlated for the REG5-RAND5 condition (see Figure S9b, REG5-RAND5: $r = .65$, $BF_{10} = 61.93$, $p < .001$, 95% CI = [0.33, 0.83]). However, for REG5a-REG5b, the test was inconclusive (REG5a-REG5b: $r = .32$, $BF_{10} = 0.74$, $p = .132$, 95% CI = [-0.10, 0.64]). Correlations increased when all trials were considered (All: $r = .75$, $BF_{10} > 1000$, $p < .001$, 95% CI = [0.59, 0.85]).

Some transitions were rated as more salient than the others (see Figure S9b and compare conditions REG5-RAND5 and REG5a-REG5b). These judgements coarsely reflected pupil dilation responses (see time-series pupil trace examples for transitions that lead to the lowest and highest total saliency in Figure S9b).

Beyond visual descriptions, we examined how saliency judgements relate to pupil

Figure S9

Saliency of transitions.

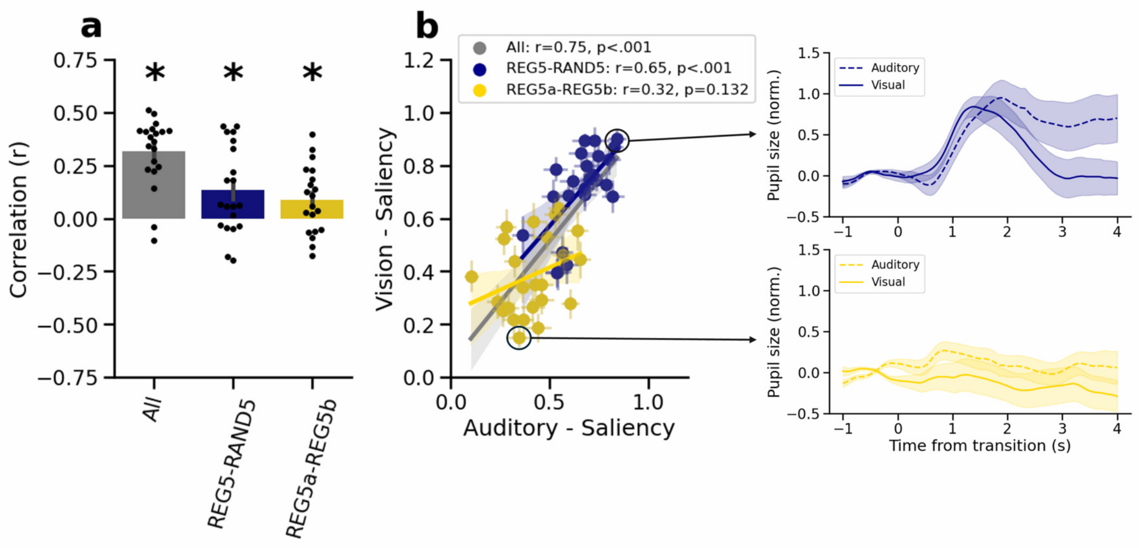

Note. The relationship between participants' saliency judgements in Experiment 3. **(a)** Correlations for participants across modalities, **(b)** the group. Pupil trace examples for transitions at the lowest and highest total saliency across modalities. Shaded areas and error bars indicate standard error of the means.

responses after transitions. We used linear regression to account for several other variables that could influence pupil size, such as baseline pupil size, transition time, trial order, sensitivity (d'), reaction times, and mental effort. This was necessary to ensure that these factors did not confound our results. This analysis also helped to reveal the relationships between the experimental variables.

Our analysis was based on all trials without separating transitions, as participants rated salience in an ordered relationship within the same session, independent of conditions (For completeness, we also reran the analysis for conditions separately in Figure S11).

Pupil responses were positively associated with participants' post-experiment saliency judgements across time, in a way that was similar across visual and auditory conditions (see Figure S10a). Baseline pupil size had a strong early effect just after the transition, which decreased gradually over time (see Figure S10b). Based on this finding, we divided the data into blocks. We found that pupil size decreased more in the visual than in the auditory REG5a-REG5b condition.

Among task-related variables, transition time tended to decrease pupil size, especially in the visual modality (Figure S10c). Pupil responses also decreased slightly throughout the experiment as trials progressed, suggesting a possible effect of habituation (Figure S10d).

d' was not related to pupil responses in either modality (Figure S10e). RTs showed weak associations with pupil size, slightly stronger in the visual condition, although these effects were small and short-lived (Figure S10f). Perceived mental effort did not show a consistent relationship with pupil responses; coefficients stayed close to zero throughout the time window (Figure S10g).

Finally, the intercept term increased after the transition in both modalities, indicating that some variability in pupil responses remained unexplained by the included predictors (Figure S10h).

Figure S10

Pupil dilation responses and experimental variables.

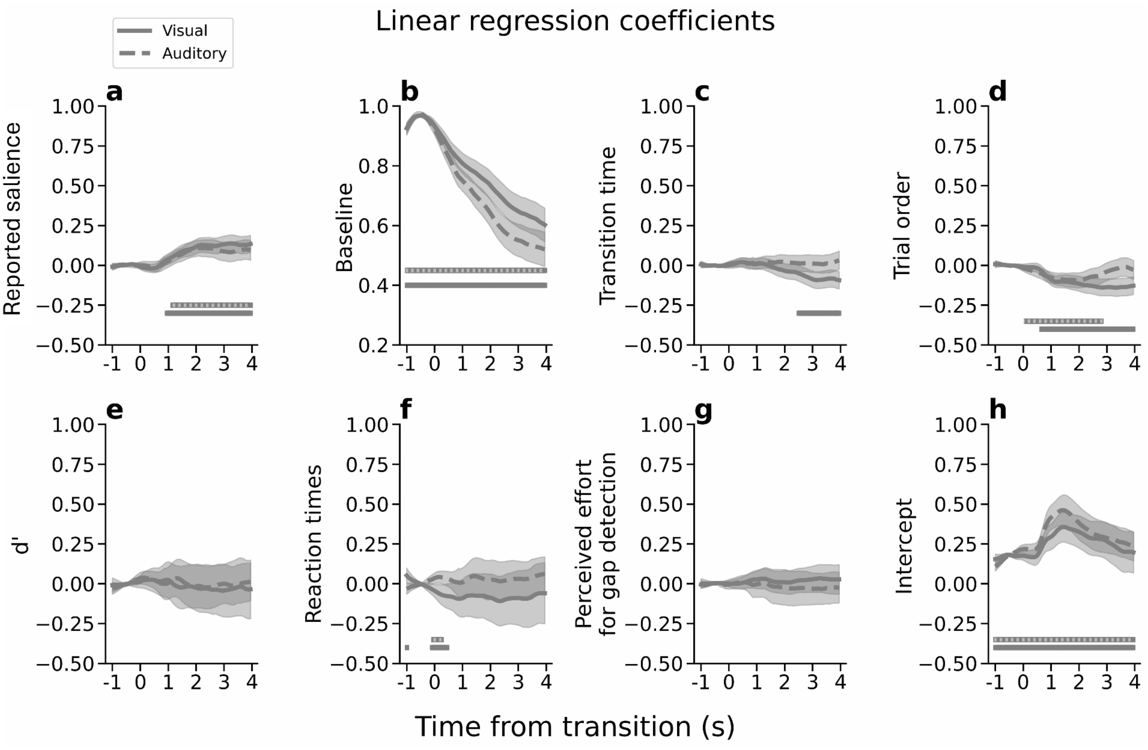

Note. Time-dependent coefficients of the linear model developed for Experiment 3. **(a)** Relationship between participants' post-experiment saliency judgements and their pupil responses over time, showing similar patterns across visual and auditory modalities. **(b)** Contribution of baseline pupil size (averaged over the 1 s before transition) to the observed pupil response. **(c)** Influence of transition time on pupil size, indicating a negative relationship between the time that transition occurs in a trial and pupil size. The effect is particularly evident for the visual modality. **(d)** Effect of trial order, showing a gradual decline in pupil response with repeated trials. **(e)** Relationship between sensitivity (d') and pupil response over time, with no consistent difference across modalities. **(f)** Effect of reaction times on pupil responses, suggesting slightly stronger associations in the visual condition. However, note that the effect is small. **(g)** Influence of perceived effort for detecting gaps, showing no strong contribution. **(h)** Intercept term reflecting general trends in pupil responses after transition, independent of predictors. Shaded regions indicate the 95% confidence intervals received from the model. The grey solid and dashed lines indicate time points where $p < .05$ for the visual and auditory modalities, respectively. No reliable difference was observed across modalities in terms of experimental variables affecting pupil size.

We observed that, in line with previous distance measures (Basgol et al., 2025), saliency was positively associated with pupil dilation, confirming earlier findings (see Figure S10).

Since participants evaluated all transitions together in the same session, this may have influenced their ratings. Our correlation analysis in the main text also supported this notion. That is why we examined the relationship between these judgments and pupil size. Here, for the sake of completeness, we conducted the same analyses again, but this time splitting them into conditions.

Pupil responses were positively associated with saliency reports of participants, with coefficients rising after the transition (Figure S11a). However, the coefficients were not statistically significant. Coefficients estimated for the REG5-RAND5 condition seem to reach significance. Baseline pupil size was a strong predictor, showing a large positive coefficient at the moment of transition that decayed rapidly over the following 4 seconds (Figure S11b).

Transition time showed a small, transient negative association with pupil size after the transition, an effect that was slightly more pronounced in the visual modality of the REG5a-REG5b condition (Figure S11c). Among the other predictors, trial order had a negative influence on pupil size, particularly in the REG5a-REG5b condition, suggesting an effect of habituation over the course of the experiment (Figure S11d).

Performance-related metrics were not strongly associated with pupil responses. d' showed only a minor relationship with pupil size, with coefficients remaining at zero after the transition (Figure S11e). Similarly, RTs and (Figure S11f) perceived effort for the gap detection task did not explain considerable variance (Figure S11g).

Finally, the intercept term showed a robust increase following the transition in all conditions, peaking around 2 seconds before slowly decaying (Figure S11h). This indicates a significant portion of the pupillary response to the transition itself remained after accounting for all other predictors in the model.

S10 Eye Events

S10.1 Saccades and blinks

Saccades and blinks were detected using built-in eye-tracker classification algorithm of the EyeLink 1000 system. Their rates were computed by identifying event onsets and evaluating frequency within a sliding window similar to pupil event rates. To assess how these

Figure S11

Pupil dilation responses and experimental variables.

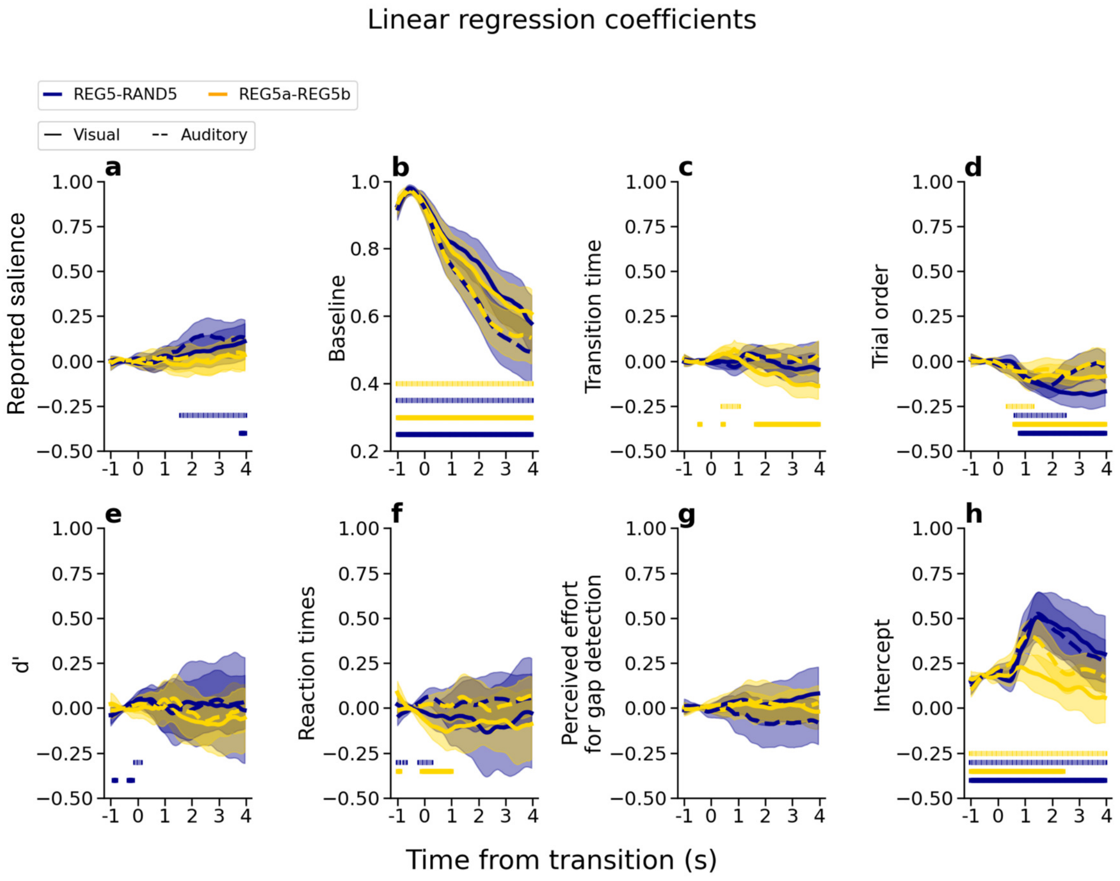

Note. Time-dependent coefficients of the linear model developed for Experiment 3. This time, we split conditions. **(a)** Relationship between participants' post-experiment saliency judgements and their pupil responses over time. **(b)** Contribution of baseline pupil size (averaged over the 1 s before transition) to the observed pupil response. **(c)** Influence of transition time on pupil size. **(d)** Effect of trial order, showing a gradual decline in pupil response with repeated trials. **(e)** Relationship between sensitivity (d') and pupil response over time. **(f)** Effect of reaction times on pupil responses. **(g)** Influence of perceived effort for detecting gaps. **(h)** Intercept term reflecting general trends in pupil responses after transition, independent of predictors. Shaded regions indicate the 95% confidence intervals received from the model. The coloured solid and dashed lines indicate time points at which $p < .05$ for the visual and auditory modalities, respectively, for specific conditions.

events evolve after transitions, we applied a 500-ms sliding window (based on each participant's mean across trials) to the event data, separately for each participant and condition. Baseline correction was applied to all rate measures to control for inter-participant variability and pre-transition fluctuations.

The mean saccade onset rate during auditory trials was approximately 0.392 Hz (SEM = 0.075), while during visual trials it was slightly lower, at approximately 0.385 Hz (SEM = 0.075). In contrast, blink rates showed a more marked difference across modalities. During auditory trials, the blink onset rate averaged around 0.016 Hz (SEM = 0.004), whereas in visual trials it was only 0.009 Hz (SEM = 0.003). There were no difference observed in terms of saccades and blink rates across conditions (Figures S12a-d).

S10.2 Microsaccades

The EyeLink 1000 system cannot detect microsaccades properly. Therefore, microsaccades were detected with millisecond accuracy using the Engbert and Kliegl velocity-threshold algorithm (Engbert and Kliegl, 2003; Schwetlick et al., 2025), which computes velocity over time and applies a threshold to identify transient fixational saccades (velocity threshold = 5, minimum microsaccade duration: 3 ms).

To estimate microsaccade rate as a continuous function over time (Figure S12), a causal kernel-based convolution was employed. Specifically, the estimated rate function $r(t)$ is obtained by convolving a microsaccadic response function $\rho(t)$ with a causal filter kernel $w(\tau)$, as follows: $r_{\text{approx}}(t) = \int_{-\infty}^{+\infty} w(\tau)\,\rho(t-\tau)\,\mathrm{d}\tau$. The response function $\rho(t)$ represents the series of microsaccade onset times as a sum of Dirac delta functions: $\rho(t) = \sum_{i=1}^{N} \delta(t - t_i)$. The causal kernel $w(\tau)$ used in this analysis is defined as: $w(\tau) = \left[\alpha^2 \tau \exp(-\alpha\tau)\right]_+$ where $\alpha = \frac{1}{30}$, corresponding to a time constant of 30 ms. The operator $[\cdot]_+$ ensures that the kernel evaluates to zero for negative time lags, preserving causality. This kernel-based method enables temporally smoothed rate estimation. The rate function $r(t)$ was computed by averaging across all trials within a participant and modality. There were no difference observed in terms of microsaccades across conditions (Figures S12e and S12f).

Figure S12

Microsaccade, saccade, and blink rates in Experiment 3.

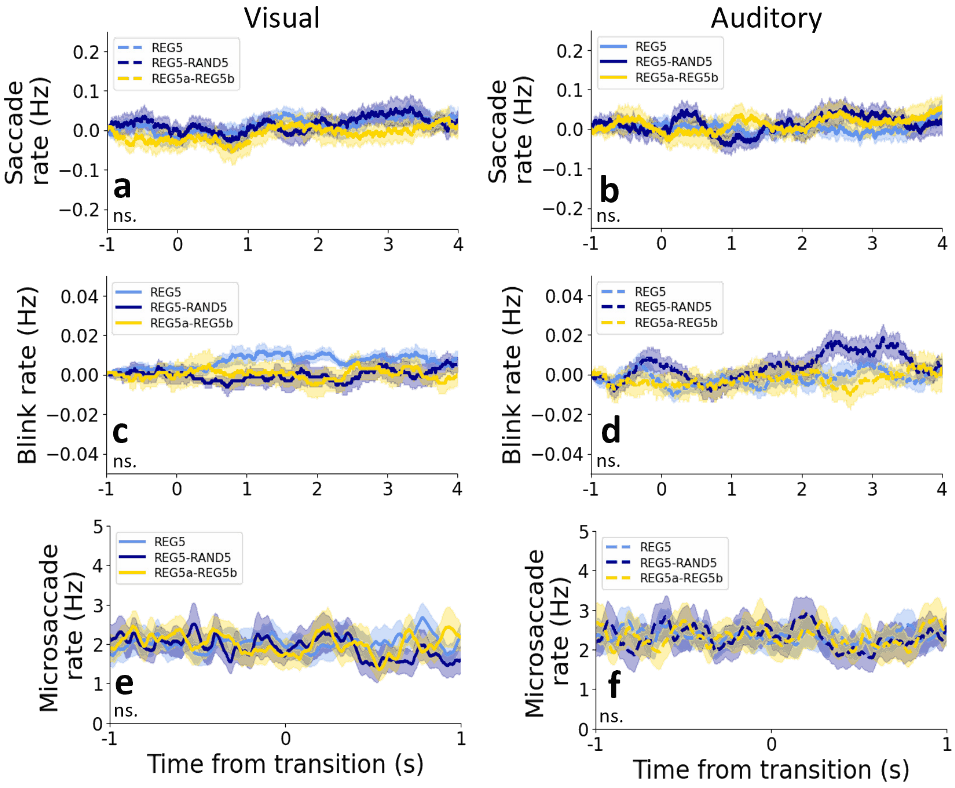

Note. Time course of event-related eye metrics in response to experimental transitions during Experiment 3. Each subplot shows condition-wise mean rates over time and shaded areas indicate between-participant standard error of the means, aligned to the transition onset (0 s). Saccade rates in the **(a)** auditory and **(b)** visual modalities. Blink rates in the **(c)** auditory and **(d)** visual modality. Microsaccade rates in the **(c)** auditory and **(d)** visual modality. Solid lines represent visual trials, and dashed lines indicate auditory trials. Conditions led to similar behaviours across conditions.

References

Akdoğan, B., Balcı, F., & Van Rijn, H. (2016). Temporal expectation indexed by pupillary response. *Timing & Time Perception*, *4*(4), 354–370. https://doi.org/10.1163/22134468-00002075

Basgol, H., Dayan, P., & Franz, V. H. (2025). Violation of auditory regularities is reflected in pupil dynamics. *Cortex*, *183*, 66–86.

Conway, C. M., & Christiansen, M. H. (2005). Modality-constrained statistical learning of tactile, visual, and auditory sequences. *Journal of Experimental Psychology: Learning, Memory, and Cognition*, *31*(1), 24–39. https://doi.org/10.1037/0278-7393.31.1.24

Éltetö, N., Nemeth, D., Janacsek, K., & Dayan, P. (2022). Tracking human skill learning with a hierarchical Bayesian sequence model. *PLoS Computational Biology*, *18*(11), e1009866.

Engbert, R., & Kliegl, R. (2003). Microsaccades uncover the orientation of covert attention. *Vision Research*, *43*(9), 1035–1045. https://doi.org/10.1016/s0042-6989(03)00084-1

Fink, L., Simola, J., Tavano, A., Lange, E., Wallot, S., & Laeng, B. (2024). From pre-processing to advanced dynamic modeling of pupil data. *Behavior Research Methods*, *56*(3), 1376–1412.

Guy, M. W., Conte, S., Bursalıoğlu, A., & Richards, J. E. (2021). Peak selection and latency jitter correction in developmental event-related potentials. *Developmental Psychobiology*, *63*(7), e22193. https://doi.org/10.1002/dev.22193

Joshi, S., Li, Y., Kalwani, R. M., & Gold, J. I. (2016). Relationships between pupil diameter and neuronal activity in the locus coeruleus, colliculi, and cingulate cortex. *Neuron*, *89*(1), 221–234. https://doi.org/10.1016/j.neuron.2015.11.028

Liao, H.-I., Kidani, S., Yoneya, M., Kashino, M., & Furukawa, S. (2016). Correspondences among pupillary dilation response, subjective salience of sounds, and loudness. *Psychonomic Bulletin & Review*, *23*, 412–425.

McLaughlin, D. J., Zink, M. E., Gaunt, L., Reilly, J., Sommers, M. S., Van Engen, K. J., & Peelle, J. E. (2023). Give me a break! Unavoidable fatigue effects in cognitive pupillometry. *Psychophysiology*, *60*(7), e14256. https://doi.org/10.1111/psyp.14256

Relaño-Iborra, H., Wendt, D., Neagu, M. B., Kressner, A. A., Dau, T., & Bækgaard, P. (2022). Baseline pupil size encodes task-related information and modulates the

task-evoked response in a speech-in-noise task. *Trends in Hearing*, *26*. https://doi.org/10.1177/23312165221134003

Schwetlick, L., Graupner, H., Dimigen, O., & Engbert, R. (2025). Distinctive pupil and microsaccade-rate signatures in self-recognition. *Journal of Vision*, *25*(4), 16. https://doi.org/10.1167/jov.25.4.16

Seabold, S., & Perktold, J. (2010). Statsmodels: Econometric and statistical modeling with Python, 92–96.

Teh, Y. W. (2006). A hierarchical Bayesian language model based on Pitman-Yor processes. *Proceedings of the 21st International Conference on Computational Linguistics and 44th Annual Meeting of the Association for Computational Linguistics*, 985–992.

Van der Wel, P., & Van Steenbergen, H. (2018). Pupil dilation as an index of effort in cognitive control tasks: A review. *Psychonomic Bulletin & Review*, *25*(6), 2005–2015. https://doi.org/10.3758/s13423-018-1432-y

Wang, L., Li, C., Chen, D., Lv, X., Go, R., Wu, J., & Yan, T. (2021). Hemodynamic response varies across tactile stimuli with different temporal structures. *Human Brain Mapping*, *42*(3), 587–597. https://doi.org/10.1002/hbm.25243
